# RIN4 fragments released by AvrRpt2 promote NDR1-dependent activation of RPS2

**DOI:** 10.1093/plcell/koag204

**Published:** 2026-07-07

**Authors:** Ahmed J Afzal, Maheen Alam, Jianhua Huang, Moneeza Akbar Agha, Luis da Cunha, Muneeza Iqbal Rai, Jibran Tahir, Anmbreen Jamroze, He Zhao, Jonathan D G Jones, David Mackey

**Affiliations:** Department of Horticulture and Crop Science, The Ohio State University, Columbus, OH 43210, United States; Department of Biology, Lahore University of Management Sciences, Sector U, DHA, Lahore 54792, Pakistan; Biology Program, NewYork University Abu Dhabi, Abu Dhabi, UAE; Department of Horticulture and Crop Science, The Ohio State University, Columbus, OH 43210, United States; The Sainsbury Laboratory, Norwich Research Park, Colney Lane, Norwich NR4 7UH, United Kingdom; The Sainsbury Laboratory, Norwich Research Park, Colney Lane, Norwich NR4 7UH, United Kingdom; John Innes Center, Norwich Research Park, Colney Lane, Norwich NR4 7UH, United Kingdom; Department of Biology, Lahore University of Management Sciences, Sector U, DHA, Lahore 54792, Pakistan; Interdisciplinary Biological Sciences Program, Northwestern University, Evanston, IL 60208, United States; Department of Horticulture and Crop Science, The Ohio State University, Columbus, OH 43210, United States; Corteva AgriSciences, DowDuPont Agriculture Division, Indianapolis, IN 46268, United States; Department of Biology, Lahore University of Management Sciences, Sector U, DHA, Lahore 54792, Pakistan; Department of Biology, Lahore University of Management Sciences, Sector U, DHA, Lahore 54792, Pakistan; Hill Labs, Parnell, Auckland 1052, New Zealand; Department of Biology, Lahore University of Management Sciences, Sector U, DHA, Lahore 54792, Pakistan; Department of Pharmacology and Therapeutics, Roswell Park Comprehensive Cancer Center, Buffalo, NY 14203, United States; The Sainsbury Laboratory, Norwich Research Park, Colney Lane, Norwich NR4 7UH, United Kingdom; The Sainsbury Laboratory, Norwich Research Park, Colney Lane, Norwich NR4 7UH, United Kingdom; Department of Horticulture and Crop Science, The Ohio State University, Columbus, OH 43210, United States; Department of Molecular Genetics, The Ohio State University, Columbus, OH 43210, United States

## Abstract

Plant nucleotide-binding, leucine-rich-repeat (NLR) immune receptors recognize pathogen effectors and activate immunity. The NLR Resistance to *Pseudomonas syringae*2 (RPS2) recognizes Avirulence protein interacting with RPS2 (AvrRpt2), a *Pseudomonas* effector that promotes virulence by proteolytically cleaving a membrane-tethered host protein, RPM1-interacting protein 4 (RIN4). RIN4 cleavage by AvrRpt2 also activates RPS2. A model in which RPS2 is activated by elimination of RIN4 is consistent with the ectopic activity of RPS2 in plants lacking RIN4 but does not explain the link between AvrRpt2's virulence activity and RPS2 activation. We found that non–membrane-tethered RIN4 derivatives are potent cytosolic activators of RPS2. Activation of RPS2 by these RIN4 derivatives, like AvrRpt2-induced activation,and unlike ectopic activation in the absence of RIN4, requires the defense signaling protein NON-RACE-SPECIFIC DISEASE RESISTANCE 1 (NDR1). Cleavage products of RIN4 produced by AvrRpt2 play contrasting roles in the activation of RPS2, with the membrane-tethered C-terminal fragment suppressing RPS2 and the non–membrane-tethered internal fragment, dependent on compatibility with the C-terminal fragment, overcoming its suppression of RPS2.

## Teaser

The bacterial effector AvrRpt2 proteolyzes the RIN4 immune hub into soluble and membrane-tethered fragments that coordinate with the NDR1 immune regulator to activate the RPS2 NLR immune receptor.

## Introduction

Plants are attacked by diverse pathogens that must overcome plant innate immunity to cause disease. Activation of immunity relies on receptors that detect pathogen-related molecules either inside or outside the plant cell. Plants utilize cell surface pattern recognition receptors (PRRs) to activate pattern-triggered immunity (PTI) upon recognition of conserved microbial features known as microbe or pathogen associated molecular patterns (MAMPs or PAMPs) (Nürnberger and Brunner 2002; Chisholm et al. 2006; da Cunha et al. 2006; Jones and Dangl 2006; Zipfel 2014). Gram-negative bacteria utilize a specialized needle-like type 3 secretion system (TTSS) to deliver effector proteins into host cells to promote effector-triggered susceptibility (ETS) by suppressing host immunity—for example, PTI—and promoting a nutritious water-rich apoplast environment (Cornelis and Van Gijsegem 2000; Alfano and Collmer 2004; Jones and Dangl 2006; Dangl et al. 2013; Gentzel et al. 2022; Roussin-Léveillée et al. 2024). Effector detection by plant NLRs activates a rapid immune response called effector-triggered immunity (ETI) that often includes localized cell death called the hypersensitive response (HR) (Heath 2000; Jones and Dangl 2006; Dangl et al. 2013). It is now known that PTI and ETI work synergistically to provide more effective resistance, highlighting the importance of mutual potentiation of cell surface and intracellular receptors for effective plant defense (Ngou et al. 2021; Chang et al. 2022).

NLRs contain central nucleotide binding and C-terminal leucine rich repeat domains and typically N-terminal coiled coil (CNLs) or toll-interleukin receptor (TNLs) domains (Hammond-Kosack and Jones 1997; Elmore et al. 2011; Bonardi et al. 2012; Contreras et al. 2023). Activation of an NLR by its corresponding effector can occur either through a direct interaction, sometimes with an integrated domain acting as a decoy of the effector's actual virulence target, or indirectly through an effector-induced modifications of a bona fide host target or a decoy (Jones and Dangl 2006; Mackey and McFall 2006; van der Hoorn and Kamoun 2008; Ellis 2016). In the latter case, the NLR is said to “guard” the host target by monitoring for its perturbation by an effector protein (Dangl and Jones 2001). A particularly well-studied example of a bona fide virulence target that is guarded by multiple NLRs is RPM1-interacting protein 4 (RIN4).

RIN4 is a 211 amino acid, intrinsically unstructured protein that contains 2 nitrate induced (NOI) domains of unknown function and is anchored to the plasma membrane by lipidation of C-terminal cysteine residues (Takemoto and Jones 2005; Kim et al. 2005a; Afzal et al. 2013; Sun et al. 2014). RIN4 negatively regulates PTI, with overexpression attenuating PTI in *Arabidopsis thaliana* (Kim et al. 2005a, 2005b; Afzal et al. 2011). Multiple *P. syringae* T3Es, including Avirulence Protein Rpm1 (AvrRpm1), Avirulence protein B (AvrB), AvrRpt2, Hrp-Outer Protein F2 (HopF2), and Hrp-Outer protein Z3 (HopZ3), target and modify RIN4 to compromise host defenses and promote bacterial growth (Mackey et al. 2002; Mackey et al. 2003; Wilton et al. 2010; Lee et al. 2015; Redditt et al. 2019). For example, AvrB induces phosphorylation of RIN4 by RIN4-interacting protein kinase (RIPK) (Chung et al. 2011; Liu et al. 2011) at T166 within the C-terminal NOI (C-NOI) domain, which counteracts activation of PTI associated with MAMP-induced phosphorylation of another key residue of RIN4, S141, also within the C-NOI (Chung et al. 2014). Another example is the proteolytic cleavage of RIN4 by AvrRpt2 within each of the 2 NOI domains, which generates 3 fragments (AvrRpt2-cleavage products, ACP1-3) (Axtell et al. 2003; Chisholm et al. 2005; Takemoto and Jones 2005). Both the soluble ACP2 and membrane-tethered ACP3, which contain truncated N-NOI and C-NOI domains, respectively, persist following their generation by AvrRpt2 and are hyperactive suppressors of PTI relative to intact RIN4 (Afzal et al. 2011). Thus, it is well established that effector-induced perturbations of RIN4, including the NOI domains, contribute to suppression of plant defense.

In addition to regulating PTI, RIN4 also regulates ETI via interaction with and effector-induced activation of multiple, evolutionarily unrelated NLRs (Mackey et al. 2002, 2003; Kim et al. 2005a; Jeuken et al. 2009; Selote and Kachroo 2010; Chung et al. 2011; Ashfield et al. 2014; Chung et al. 2014; Mazo-Molina et al. 2020; Prokchorchik et al. 2020). A central prediction of the guard hypothesis is that the virulence-associated modification of a guardee will trigger the activation of an associated NLR (Dangl and Jones 2001). Consistent with this prediction, modifications of RIN4 induced by AvrRpm1 or AvrB activate a plasma membrane localized CNL, Resistance to *Pseudomonas syringae* pv. *maculicola* 1 (RPM1) (Chung et al. 2011). In the absence of effector activation, RIN4 interacts with and negatively regulates RPM1 at the plasma membrane (Boyes et al. 1998; Mackey et al. 2002; Belkhadir et al. 2004; Gao et al. 2011). Through perturbation of RIN4, AvrB and AvrRpm1 activate the soybean (*Glycine max*) CNLs Resistance to *Pseudomonas syringae* pv. *glycinea* b (Rpg1b) and Resistance to *Pseudomonas syringae* pv. *glycinea* r (Rpg1r), respectively, and AvrRpm1 also weakly activates another Arabidopsis CNL, Resistance to *Pseudomonas syringae*2 (RPS2) (Kim et al. 2009). RPS2, an apple CNL *Malus* × *robusta* 5 (MR5), and a tomato (*Solanum lycopersicoides*) CNL *Pseudomonas* tomato race 1 (Ptr1) each respond to proteolysis of RIN4 by homologs of AvrRpt2 (Mackey et al. 2003; Mazo-Molina et al. 2020; Prokchorchik et al. 2020). Remarkably, *RPM1*, *Rpg1b*, *Rpg1r*, *RPS2*, *MR5*, and *Ptr1* each evolved independently (Ashfield et al. 2014; Mazo-Molina et al. 2020; Prokchorchik et al. 2020). Collectively, these findings highlight the importance of “guarding” RIN4 for plant immunity.

The mechanism underlying how virulence-associated proteolysis of RIN4 by AvrRpt2 activates RPS2 remains obscure. In Arabidopsis plants lacking RIN4, RPS2 is ectopically active, that is, active in the absence of an activating T3E (Mackey et al. 2003; Day et al. 2005). Thus, a simple interpretation is that elimination of RIN4 by AvrRpt2 activates RPS2 (Axtell and Staskawicz 2003; Mackey et al. 2003; Day et al. 2005). However, unlike ectopic activation of RPS2, activation of RPS2 by AvrRpt2 requires the RIN4-interacting protein NDR1 (NON-RACE-SPECIFIC DISEASE RESISTANCE1) (Century et al. 1995; Tornero et al. 2002; Belkhadir et al. 2004; Day et al. 2006). MR5 is not ectopically active and is activated by the ACP3 fragment of apple RIN4 (Prokchorchik et al. 2020). The ACP2 and ACP3 fragments, which are defense-suppressive in Arabidopsis, are candidates for involvement in the NDR1-dependent activation of RPS2.

We previously observed that RIN4 derivatives not tethered to the plasma membrane, because they lack the C-terminal fatty acylation motif, are more potent suppressors of PTI and also elicit an HR-like cell death response (Afzal et al. 2011). We speculated that these non–membrane-tethered derivatives of RIN4 might elicit an HR by functioning in a manner analogous to the soluble ACP2 fragment produced by AvrRpt2. Here we show that non–membrane-tethered derivatives of RIN4, which localize to both the cytosol and nucleus, function in the cytosol to activate RPS2. Similar to AvrRpt2-dependent activation, RPS2 activation by non–membrane-tethered derivatives of RIN4 requires NDR1. ACP2 and ACP3 play contrasting roles in RPS2 regulation. Membrane-tethered ACP3 retains the ability to inhibit ectopic activation of RPS2. Conversely, non-membrane-tethered ACP2, which localizes to both the cytosol and nucleus, functions outside the nucleus to activate RPS2 by overcoming suppression mediated by ACP3. NDR1 recruits ACP2 to the proximity of RPS2, and compatibility between ACP2 and ACP3 is required for ACP2 to overcome suppression by ACP3, indicating that ACP2 likely interacts with a pre-activation complex containing RPS2, ACP3, and NDR1. Consistent with predictions based on the “guard hypothesis”, these findings link the virulence-associated perturbation of RIN4 by AvrRpt2 to the activation of RPS2.

## Results

### Cell death caused by non–membrane-tethered derivatives of RIN4 is dependent on RPS2

**Figure 1:**
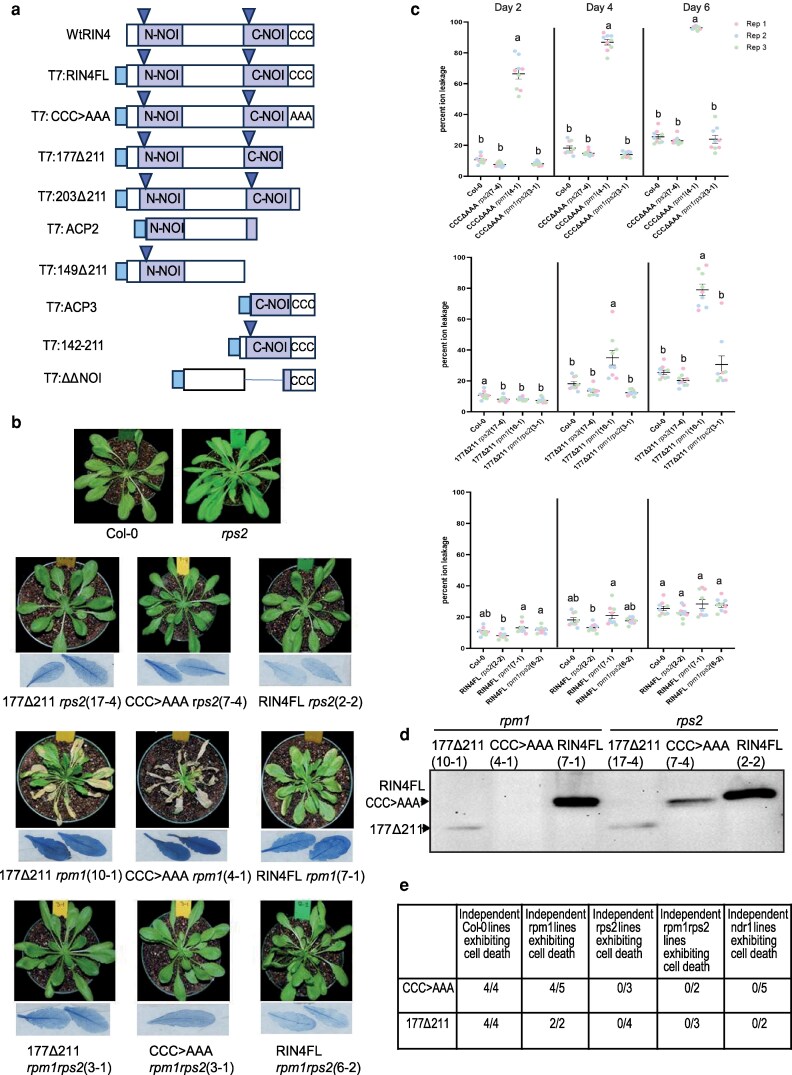
Non–membrane-tethered derivatives of RIN4 cause an RPS2-dependent cell death response in Col-0. a) RIN4 derivatives expressed from a Dex-inducible promoter in transgenic Arabidopsis plants. Purple rectangle, NOI-domain; blue triangle, site of cleavage by AvrRpt2; light blue square, T7-tag; RIN4FL, RIN4 full length. b) Top panel: Macroscopic symptoms were observed at 72 h post induction (HPI) with dexamethasone (dex) of indicated RIN4 derivatives in homozygous transgenic Arabidopsis plants. Panel below HR images: Trypan blue staining of leaves from plants as in B. c) Cell death in plants as in B was quantified by measuring electrolyte leakage. Data were collected from 3 independent experiments with 3 technical replicates per transgenic line (n = 9). Error bars represent SEM. Statistical analysis was performed using 1-way ANOVA, followed by Tukey multiple comparison test. d) Anti-T7 immunoblot showing protein accumulation of CCC > AAA and 177Δ211 in *rpm1* or *rps2* at 72 HPI. Arrows indicate positions of individual RIN4 derivatives. e) Occurrence of cell death following induction of non–membrane-tethered RIN4 derivatives in additional T2 lines of Col-0, *rpm1*, *rps2*, *rpm1rps2*, or *ndr1*.

RIN4 contains a C-terminal fatty acylation site that includes 3 cysteines at residues 203 to 205 (Takemoto and Jones 2005). Palmitoylation of 1 or more of these C-terminal cysteines causes plasma membrane localization of RIN4 (Takemoto and Jones 2005; Kim et al. 2005a). Three non–membrane-tethered derivatives of RIN4 (177Δ211, 203Δ211, and CCC > AAA) that lack the C-terminal acylation site (Fig. 1a) elicit a macroscopic cell death response when expressed as transgenes under control of a dexamethasone (dex)-inducible promoter in the *Arabidopsis* ecotype Col-0 (Fig. S1a and b). The strong cell death response elicited by non–membrane-tethered RIN4 derivatives cannot be attributed to expression levels; anti-T7 immunoblotting demonstrated that full-length RIN4 (RIN4FL) and 149Δ211, which induce only modest chlorotic symptoms, accumulate to significantly higher levels than the non–membrane-tethered RIN4 derivatives (Fig. S1c) (Afzal et al. 2011).

Given its similarity to an HR, we reasoned that the cell death elicited by these non–membrane-tethered derivatives likely results from the activation of either RPM1 and/or RPS2. To test this prediction, transgenic lines expressing dex-inducible RIN4FL, 177Δ211, and CCC > AAA were established in *rpm1-3* or *rps2-101C* single mutant or *rpm1rps2* double mutant Arabidopsis plants. Similar to expression of 177Δ211 or CCC > AAA in *rpm1rps2rin4* triple mutant Arabidopsis plants (Afzal et al. 2011), expression of these derivatives in either *rps2* single mutant or *rpm1rps2* double mutant plants did not elicit a cell death response (Fig. 1b and c). However, their expression in *rpm1* single mutant plants elicited a cell death response (Fig. 1b and c). The difference in the cell death responses could not be attributed to the expression levels of CCC > AAA and 177Δ211 in different transgenic lines (Fig. 1d). While accumulation of 177Δ211 was detectable in both *rpm1* and *rps2* plants, CCC > AAA could not be detected in *rpm1*, likely due to the stronger cell death response it causes compared with 177Δ211. We tested the response in additional, independent transgenic lines and observed that expression of CCC > AAA and177Δ211 in plants expressing RPS2 (Col-0 or *rpm1*) elicited cell death in 8 out of 9 and 6 out of 6 lines, respectively, whereas cell death was observed in none of the 12 lines in *rps2* or *rpm1rps2* (Fig. 1e). Collectively, these results indicate that activation of cell death by non-membrane-tethered RIN4 derivatives is RPS2-dependent and is not suppressed by native RIN4.

### The NOI domains mediate both suppression of RPS2 within membrane-tethered derivatives of RIN4 and activation of RPS2 within non–membrane-tethered derivatives of RIN4

Next, we sought to determine the contribution of NOI domains within membrane-tethered derivatives of RIN4, Flag:1Δ64 (lacks N-NOI), Flag:149Δ176 (lacks C-NOI), or Flag:ΔΔNOI (double deletion of 1Δ64 and 149Δ176, lacks both NOI domains), toward suppression of RPS2 (Fig. S2a). Consistent with previous observations (Day et al. 2005), Flag:1Δ64 suppressed RPS2 as effectively as Flag:RIN4FL in *N. benthamiana* (Fig. S2b). Despite accumulating to similar levels as RIN4FL, Flag:149Δ176 modestly and Flag:ΔΔNOI minimally suppressed RPS2 (Fig. S2b and c). Thus, the NOI domains in the membrane-tethered derivatives are required for RPS2 suppression in transient assays.

To further assess the role of NOI domains within non–membrane-tethered derivatives of RIN4 in overcoming suppression of RPS2 by native RIN4, we generated Arabidopsis lines that inducibly express additional derivatives of RIN4 (Fig. 1a). Lines expressing derivatives that contain both the NOIs (CCC > AAA, 203Δ211 and 177Δ211) displayed strong cell death, while those expressing derivatives lacking the C-NOI domain displayed weaker (ACP2) or no (149Δ211) cell death (Fig. S1a and b). The reduced activity of ACP2 and 149Δ211 did not result from lack of expression; they accumulated to significantly higher levels than the cell death–inducing derivatives with the C-NOI, which were barely or not detectable, likely due to the induced cell death (Fig. S1c). None of the plants expressing membrane-tethered derivatives of RIN4, including ACP3 (AA153-211, with a truncated C-NOI domain), 142–211 (containing an intact C-NOI domain), or ΔΔNOI (lacking both NOI domains) displayed cell death despite readily detectable protein accumulation (Fig. S3a–c). However, the membrane-tethered derivatives with either a truncated (ACP3) or full (142–211) C-NOI produced chlorotic symptoms resembling those observed with RIN4FL and ACP2 (Fig. S1a and S3a). Collectively, data from the transient and stable transgenic assays indicate that NOI domains mediate either activation or repression of RPS2 depending on whether they are within a non–membrane-tethered or membrane-tethered derivative of RIN4, respectively.

### Activation of RPS2 by non–membrane-tethered derivatives of RIN4 requires NDR1

NDR1 is a plasma membrane-localized, glycophosphatidyl-inositol (GPI)-anchored protein required for proper function of several R-proteins, including RPM1, RPS2, and RPS5 (Tornero et al. 2002; Belkhadir et al. 2004; Coppinger et al. 2004). In planta interaction between NDR1 and RIN4 is proposed to contribute to its role in RPM1 and RPS2 signaling (Day et al. 2006). The ectopic activation of RPS2 that underlies seedling lethality in homozygous *RPS2rin4* plants still occurs immediately following germination in *RPS2rin4ndr1* plants (Belkhadir et al. 2004). This contrasts with activation of RPS2 by AvrRpt2, which requires NDR1.

**Figure 2:**
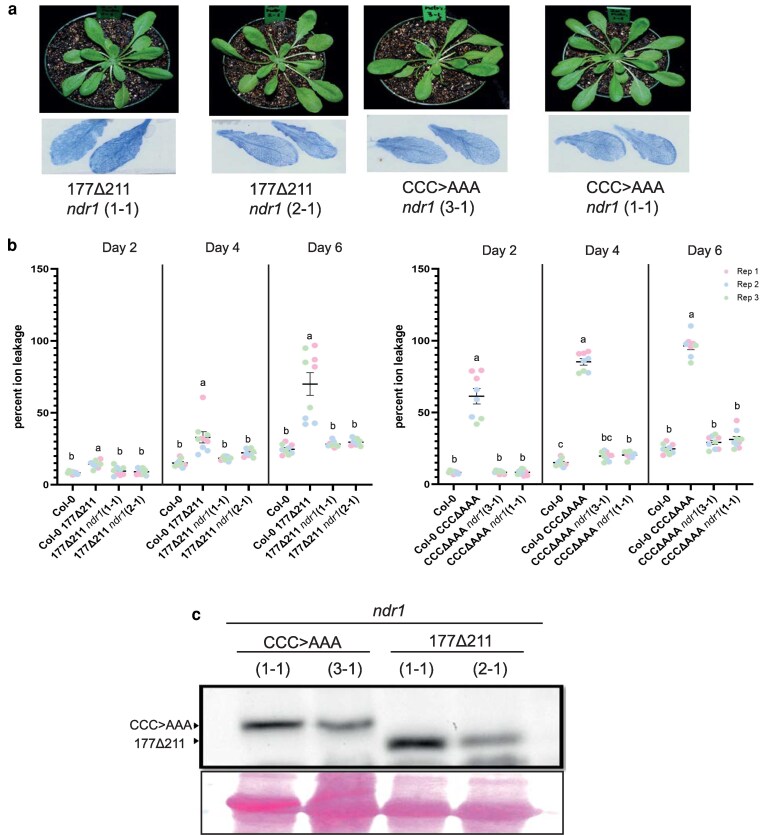
Cell death caused by the non–membrane-tethered derivatives of RIN4 is NDR1 dependent. a) Macroscopic symptoms were observed at 72 HPI with dex of indicated non–membrane-tethered RIN4 derivatives in homozygous transgenic *ndr1* plants. Panel below: Trypan blue staining of leaves from plants as in A. b) Cell death in plants as in A was quantified by measuring electrolyte leakage. Data were collected from 3 independent experiments with 3 technical replicates per transgenic line (n = 9). Error bars represent SEM. Statistical analysis was performed using 1-way ANOVA, followed by Tukey multiple comparison test. c) Anti-T7 immunoblot showing protein accumulation of CCC > AAA and 177Δ211 in *ndr1* lines at 72 HPI. The lower panel shows Ponceau staining of Rubisco as a loading control. Arrows indicate positions of individual RIN4 derivatives.

To determine the role of NDR1 in the activation of RPS2 by non–membrane-tethered RIN4 derivatives, transgenic lines carrying inducible constructs were generated in the *ndr1-1* mutant background. Expression of CCC > AAA or 177Δ211 failed to elicit cell death in *ndr1* plants despite detectable accumulation of each derivative (Fig. 2a–c). The failure of CCC > AAA and 177Δ211 to activate RPS2 in *ndr1* mutant plants was observed in 5 and 2 independent transgenic lines, respectively (Fig. 1e). Thus, like the activation of RPS2 by AvrRpt2 and unlike the ectopic activation of RPS2 in the absence of RIN4, the activation of RPS2 by non–membrane-tethered derivatives of RIN4 requires *NDR1*.

To further examine the role of AtNDR1 in ectopic and effector-induced activation of RPS2, we expressed various combinations of Flag:RIN4FL, RPS2:HA, HA:NDR1, and either AvrRpt2:HA or the proteolytically inactive mutant AvrRpt2^C122A^:HA in *N. benthamiana* leaves (Fig. S4a). As expected, the ectopic activity of RPS2:HA in the absence of Flag:RIN4FL was unaffected by the presence or absence of HA:NDR1, and coexpression of Flag:RIN4FL suppressed the activity of RPS2:HA (Fig. S4b and c). Also as expected, expression of AvrRpt2:HA, but not AvrRpt2^C122A^:HA, overcame the suppression and activated RPS2:HA (Fig. S4b). Interestingly, coexpression of HA:NDR1 attenuated activation of RPS2 by AvrRpt2 (Fig. S4b). Whether this inhibitory effect results from NDR1 accumulation at the plasma membrane (Day et al. 2006) or mis-localization due to overexpression is unclear. Collectively these data show that, as in Arabidopsis, the ectopic activity of RPS2 in *N. benthamiana* is unaffected by levels of NDR1 (Belkhadir et al. 2004). Although constitutive overexpression of AtNDR1 did not inhibit RPS2 activation by AvrRpt2 in Arabidopsis (Coppinger et al. 2004), its suppressive activity when transiently overexpressed in *N. benthamiana* further indicates its involvement in AvrRpt2-mediated activation of RPS2.

### Non–membrane-tethered derivatives of RIN4 activate RPS2 in the cytosol where they compete with intact RIN4 for association with RPS2

Unlike derivatives of RIN4 with an intact C-terminal acylation site, ACP2, 177Δ211, and CCC > AAA were not found in the endomembrane fraction in Arabidopsis (Afzal et al. 2011). When expressed in *N. benthamiana*, YFP:RIN4FL localized to the plasma membrane, whereas the YFP-tagged versions of these non–membrane-tethered derivatives of RIN4 accumulated in both the cytosol and nucleus, the latter based on their co-localization with Hoechst dye (Fig. S5a–c).

To determine whether the non–membrane-tethered derivatives of RIN4 mediate RPS2 activation in the nucleus or cytosol, derivatives of CCC > AAA carrying a nuclear localization signal (NLS, YFP:NLS:CCC > AAA), a nuclear export signal (NES, YFP:NES:CCC > AAA), or a scrambled nuclear export signal (SNE, YFP:SNE:CCC > AAA) were constructed and shown to localize as expected in the nucleus, cytosol, or both, respectively (Fig. S5a and d). When coexpressed with RPS2 and RIN4FL, the nuclear-excluded YFP:NES:CCC > AAA derivative overcame suppression by Flag:RIN4FL and activated RPS2-induced cell death at comparable levels to ectopic activation of RPS2 in *N. benthamiana*. In contrast, nuclear-localized YFP:NLS:CCC > AAA did not activate RPS2 (Fig. S5e). This result was confirmed under conditions in which agrobacteria concentrations were adjusted to give equivalent expression of both YFP:NLS:CCC > AAA and YFP:NES:CCC > AAA (Fig. S5f and g). Thus, cytosolic accumulation of the CCC > AAA derivative of RIN4 is required for its ability to activate RPS2.

**Figure 3:**
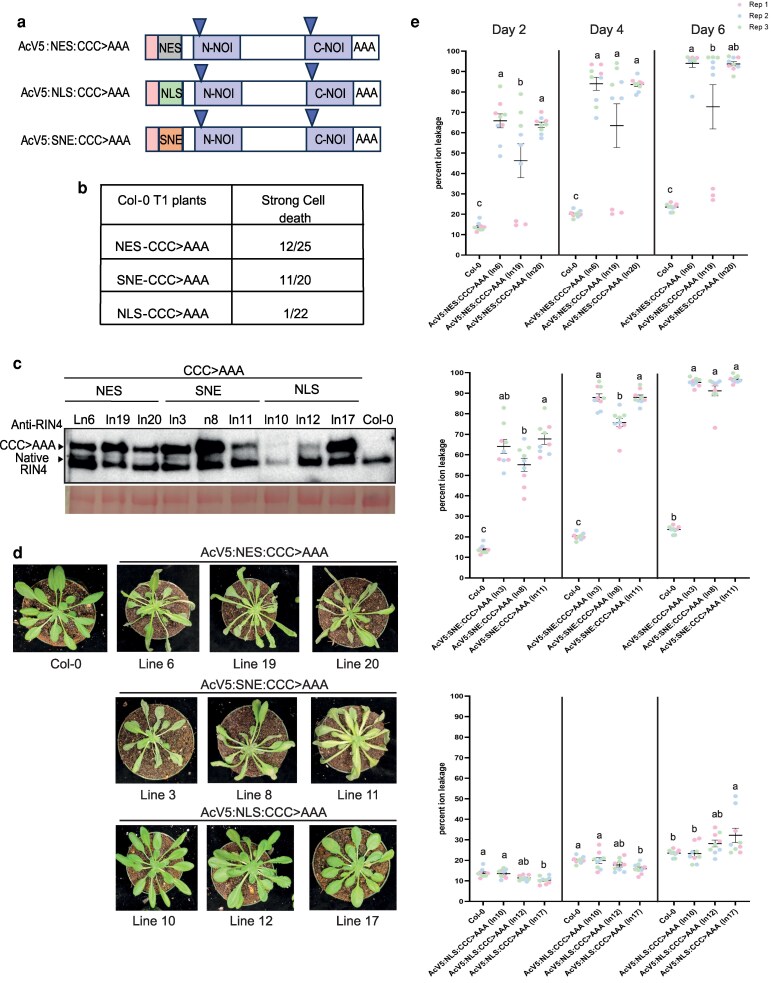
Non–membrane-tethered CCC > AAA must accumulate in the cytosol to activate RPS2 in Arabidopsis. a) RIN4 derivatives expressed from a Dex-inducible promoter in transgenic Arabidopsis plants. Peach rectangle, AcV5 tag; N-terminal square, NES, SNE, or NLS tags. b) Occurrence of symptoms at 72HPI in independent T1 lines induced to express the indicated derivatives of RIN4. c) Anti-RIN4 immunoblot showing protein accumulation of the RIN4 derivatives in the indicated homozygous transgenic Arabidopsis plants at 72 HPI. The lower panel shows Ponceau staining of Rubisco. Arrows indicate the positions of native RIN4 and the tagged-CCC > AAA derivatives. d) Macroscopic symptoms were observed at 72 HPI with dex of indicated non–membrane-tethered CCC > AAA derivative in homozygous transgenic Arabidopsis. e) Cell death in plants as in D was quantified by measuring electrolyte leakage. Data were collected from 3 independent experiments with 3 technical replicates per transgenic line (n = 9). Error bars represent SEM. Statistical analysis was performed using 1-way ANOVA, followed by Tukey multiple comparison test.

To validate results from transient assay in *N. benthamiana*, transgenic lines of Arabidopsis (Col-0) that inducibly express CCC > AAA derivatives tagged with AcV5 and either NLS, NES, or SNE were generated (Fig. 3a). When individual leaves of T1 plants were treated with dexamethasone, strong cell death was observed in only 1 of 22 plants transformed with the inducible AcV5:NLS:CCC > AAA compared with 12 of 25 and 11 of 20 with the AcV5:NES:CCC > AAA or AcV5:SNE:CCC > AAA constructs, respectively (Fig. 3b). This observation was further supported by generating and testing 3 independent transgenic lines for each construct that had variable but overlapping levels of protein expression (Fig. 3c). Lines expressing AcV5:NES:CCC > AAA or AcV5:SNE:CCC > AAA displayed strong cell death, while lines expressing AcV5:NLS:CCC > AAA did not (Fig. 3d and e).

RIN4 and RPS2 associate in planta (Axtell and Staskawicz 2003; Mackey et al. 2003). To determine if RPS2 also associates with the non–membrane-tethered CCC > AAA derivative, we generated 35S:RPS2:TurboID:V5 (Fig. S6a) to enable TurboID-based proximity labeling in *N. benthamiana*. 35S:RPS2:TurboID:V5 or Brassinosteroid-Insensitive 1 (BRI1) fused to TurboID, as a negative control, were expressed with either Flag:RIN4FL or the cytosol-localized AcV5:NES:CCC > AAA derivative. As expected, RPS2:TurboID:V5 biotinylated Flag:RIN4FL (Fig. S6b) (Axtell and Staskawicz 2003; Mackey et al. 2003) and also biotinylated AcV5:NES:CCC > AAA (Fig. S6b). The reduced labeling of AcV5:NES:CCC > AAA, relative to RIN4FL, likely results from the cell death that occurs because the AcV5:NES:CCC > AAA RIN4 derivative fails to suppress RPS2. Interestingly, labeling of Flag:RIN4FL by RPS2:TurboID:V5 was greatly diminished when AcV5:NES:CCC > AAA was coexpressed (Fig. S6c). Conversely, labeling of AcV5:NES:CCC > AAA was unaffected by coexpression of Flag:RIN4FL (Fig. S6d). These data indicate that the CCC > AAA derivative of RIN4 effectively outcompetes RIN4FL for association with RPS2. The ability of the CCC > AAA to compete with RIN4FL for association with RPS2 was tested further by co-immunoprecipitation. A derivate of RPS2 (RPS2Δ^N26^:Flag) with a truncated N terminus, which eliminates its ability to cause cell death and thus allows for better accumulation of transiently expressed proteins (Guo et al. 2026), was coexpressed with HA:RIN4FL alone or in combination with increasing amounts of AcV5:NES:CCC > AAA (Fig. S6e). As expected, HA:RIN4FL associated with RPS2Δ^N26^:Flag, but not BRI1:Flag. Consistent with the proximity labeling results, this association was disrupted in a dose-dependent manner by AcV5:NES:CCC > AAA. Despite the close proximity of AcV5:NES:CCC > AAA to RPS2 (Fig. S6d), it fails to coimmunoprecipitate with RPS2Δ^N26^:Flag, indicating that their interaction is transient or unstable. Collectively, the data indicates that non–membrane-tethered derivatives of RIN4 activate RPS2 at the plasma membrane, likely by displacing the association of inhibitory, membrane-tethered RIN4 with RPS2.

### ACP2 is associated with RPS2 to overcome ACP3-mediated suppression and activate RPS2

**Figure 4:**
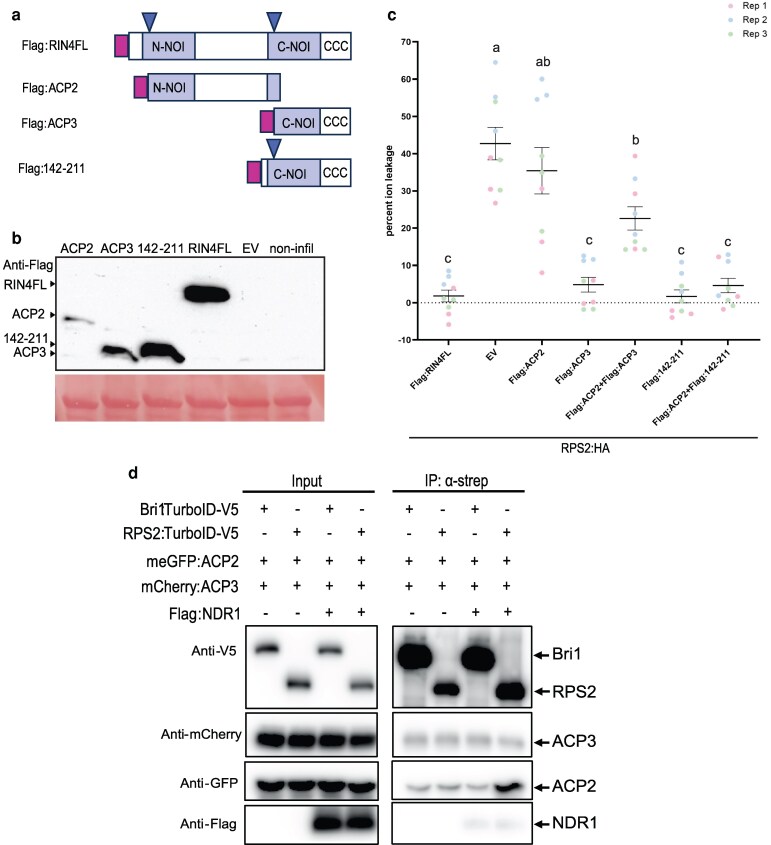
ACP2 activates RPS2 suppressed by ACP3 in *N. benthamiana*. a) RIN4 derivatives expressed from 35S promoter in transient experiments in *N. benthamiana*. Pink rectangle, Flag-tag. b) Anti-Flag immunoblot showing protein accumulation of the indicated RIN4 derivatives in *N. benthamiana* at 72 HPI. The lower panel shows Ponceau staining of Rubisco. Arrows indicate position of individual RIN4 derivatives. c) RIN4 derivatives (each at OD600 = 0.6) and RPS2-HA (at OD600 = 0.04) were infiltrated in *N. benthamiana* leaves. Cell death in plants was quantified by measuring electrolyte leakage at 96 HAI. Data were collected from 3 independent experiments with 3 technical replicates per treatment (n = 9). Error bars represent SEM. Statistical analysis was performed using 1-way ANOVA, followed by Tukey multiple comparison test. d) *N. benthamiana* leaves were infiltrated with agrobacterium containing the indicated constructs (each at OD600 = 0.5). After 24 h the leaves were infiltrated with biotin and 4 h later tissue was collected. Input and anti-strep IP samples were immunoblotted as indicated with black arrows indicating the position of the proteins.

Collectively, our data indicate that NOI domains contribute to inhibition or activation of RPS2 depending on whether they are in a membrane-tethered or cytosolic form. Furthermore, RPS2 activation by cytosolic NOI domains is dependent on NDR1. These findings led us to speculate that, during the NDR1-dependent activation of RPS2 by AvrRpt2, suppression of RPS2 by ACP3 is overcome by ACP2. To test this hypothesis, we coexpressed Flag:ACP2 and/or Flag:ACP3 along with RPS2:HA in *N. benthamiana* (Fig. 4a). Although Flag:ACP3 was expressed to lower levels than RIN4FL, both were equally competent to suppress RPS2:HA (Fig. 4b and c). As expected, Flag:ACP2 failed to suppress RPS2:HA. Consistent with our hypothesis, coexpression of Flag:ACP2 overcame suppression by Flag:ACP3 and activated RSP2:HA. Furthermore, as was observed for RPS2 activation by AvrRpt2, overexpression of NDR1:HA suppressed the ability of Flag:ACP2 to overcome suppression by Flag:ACP3 and activate RPS2:HA (Fig. S4c).

**Figure 5:**
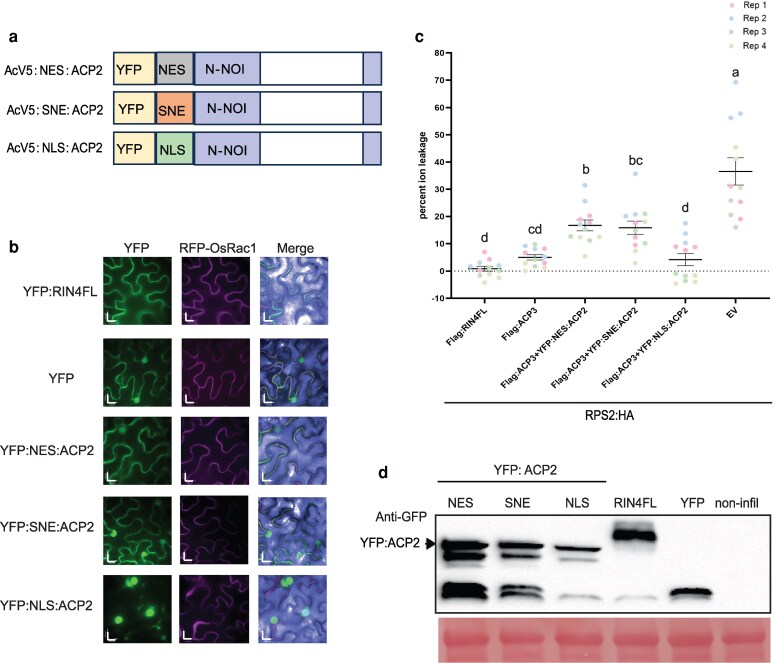
ACP2 must accumulate in the cytosol to activate RPS2 in *N. benthamiana*. a) ACP2 derivatives expressed from 35S promoter in transient experiments in *N. benthamiana*. AcV5 tags are followed by rectangles indicating the NLS, NES, or SNE tags. b) The indicated derivatives of RIN4 were coexpressed with the plasma membrane marker RFP-OsRac1 in *N. benthamiana*. Shown are YFP, RFP, and merged confocal microscope images at 72 HAI. Scale bars are 25 μM. c) RIN4 derivatives (OD600 = 0.6) and RPS2-HA (OD600 = 0.04) infiltrated in *N. benthamiana* leaves. Cell death in plants was quantified by measuring electrolyte leakage at 96 HAI. Data were gathered from 4 independent experiments with 3 technical replicates per treatment (n = 12). Error bars represent SEM. Statistical analysis was performed using 1-way ANOVA, followed by Tukey multiple comparison test. d) Anti-GFP immunoblot showing accumulation of the indicated proteins in *N. benthamiana* at 72HAI. Arrows indicate position of the RIN4 derivatives.

Since ACP2 accumulates in both the cytosol and nucleus (Afzal et al. 2011), we sought to confirm that cytosolic ACP2 activates RPS2. Derivatives of ACP2 with YFP and either NLS, NES, or SNE at their N-termini localized as expected when expressed in *N. benthamiana* (Fig. 5a and b). When these derivatives were expressed in *N. benthamiana* along with Flag:ACP3 and RPS2:HA, YFP:SNE:ACP2 or YFP:NES:ACP2, but not YFP:NLS:ACP2, activated RPS2:HA (Figs 5c). Expression levels of the proteins do not account for their (in)ability to activate ACP3-suppressed RPS2 (Fig. 5d). Thus, as for non–membrane-tethered derivatives of RIN4, ACP2 must be present in the cytosol to activate RPS2.

ACP2 associates with RPS2 dependent on access enabled by cleavage within the C-NOI. Similar to Flag:ACP3, a derivative of RIN4 with an intact C-NOI domain, Flag:142–211 also suppressed RPS2 in *N. benthamiana* (Fig. 4a). However, Flag:ACP2 failed to activate RPS2:HA suppressed by Flag:142–211 (Fig. 4c). This difference likely results from steric interference rather than a difference between an intact and cleaved C-NOI since Flag:ACP2 also failed to activate RPS2:HA suppressed by ACP3 with a bulky N-terminal adduct, YFP:ACP3 or mCherry:ACP3 (Fig. S7a–c). Also, similar to its weak activation of RPS2 suppressed by native RIN4 in Arabidopsis transgenic lines (Fig. S1), Flag:ACP2 failed to activate RPS2:HA that was suppressed by Flag:RIN4FL in *N. benthamiana* (Fig. S7a). These data support the hypothesis that ACP2 activates RPS2 dependent on access to an ACP3-RPS2 complex created upon cleavage of RIN4 within its C-terminal NOI.

We further tested this hypothesis by using TurboID to examine the proximity of ACP2 to RPS2 under various conditions (Fig. 4d). To facilitate protein accumulation before the onset of RPS2 induced HR, we used estradiol-inducible RPS2 (or BRI1) and with ACP3 with a bulky N-terminal adduct. BRI1:TurboID:V5 or RPS2:TurboID:V5 were coexpressed with meGFP:ACP2 and mCherry:ACP3, either with or without Flag:NDR1. Surprisingly, labeling of neither Flag:NDR1 nor mcherry:ACP3 was detected and labeling of meGFP:ACP2 was detected only in the presence of Flag:NDR1. Thus, even when ACP2 is unable to activate RPS2 because it is suppressed by ACP3 with a bulky C-terminal adduct, it only comes into close proximity to the ACP3-RPS2 complex dependent on expression of AtNDR1. Collectively, these results indicate that ACP2 is recruited to the RPS2 complex by NDR1 and most efficiently accesses the complex when the N terminus of ACP3 is exposed.

### Activation of RPS2 requires compatibility between ACP2 and ACP3

**Figure 6:**
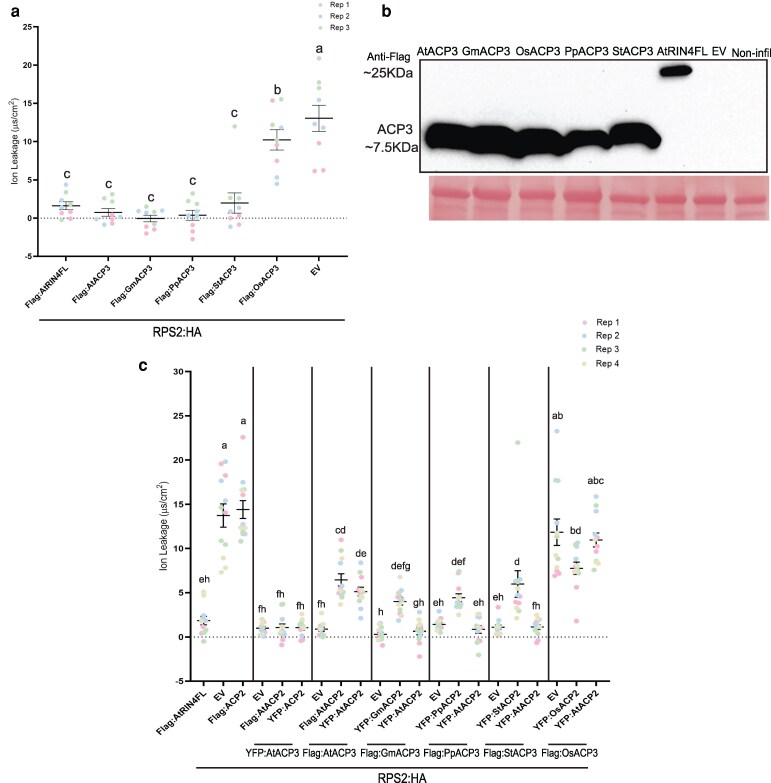
Activation of RPS2 suppressed by ACP3 requires compatibility between ACP2 and ACP3. a) RIN4 derivatives (OD600 = 1.0) and RPS2-HA (OD600 = 0.05) infiltrated in *N. benthamiana* leaves. Cell death in plants was quantified by measuring electrolyte leakage at 72 HAI. Data were collected from 3 independent experiments with 3 technical replicates per treatment (n = 9). Error bars represent SEM. Statistical analysis was performed using 1-way ANOVA, followed by Tukey multiple comparison test. b) Anti-Flag immunoblot shows accumulation of Flag-RIN4 derivatives expressed in *N. benthamiana* at 72 HAI. The lower panel shows Ponceau staining of Rubisco. c) Electrolyte leakage at 96 HAI for agro-transient expression of 4 independent experiments with 3 technical replicates per treatment (n = 12). Error bars represent SEM. Statistical analysis was performed using 1-way ANOVA, followed by Tukey multiple comparison test.

We previously demonstrated that C-terminal fragments equivalent to ACP3 (YFP:RIN4^CLV3^, referred to as YFP:ACP3 in the current study) from RIN4 homologs of soybean, apple, peach, and potato, but not rice, are capable of suppressing RPS2:HA when transiently expressed in *N. benthamiana* (Alam et al. 2021). To examine the ability of ACP2-equivalent fragments to activate RPS2 that was suppressed by RIN4 homologs from other plants, we generated Flag-tagged versions of ACP3 without the bulky, N-terminal YFP-tag. As for the YFP-tagged versions, Flag:ACP3 derivatives from soybean, peach, or potato, but not rice, suppressed RPS2 (Fig. 6a and b and Fig. S8). As expected, RPS2:HA suppressed by Flag:AtACP3, but not YFP:AtACP3, was activated by Flag:AtACP2 as well as a related derivative, YFP:AtACP2, that lacks ACP1 and the N-terminal RIN4 cleavage site (Fig. 6c). Surprisingly, YFP:AtACP2 failed to activate RPS2:HA that was suppressed by the Flag:ACP3 derivatives from soybean, peach, or potato. However, RPS2:HA suppressed by these Flag:ACP3 derivatives was activated by the YFP:ACP2 derivatives from the corresponding plant species (Fig. 6c). The necessary compatibility between ACP2 and ACP3 indicates that, following their generation upon cleavage by AvrRpt2, these fragments may directly interact during activation of RPS2.

## Discussion

NLRs activate defense upon effector recognition by diverse mechanisms. Within a phylogenetic tree of CNLs, RPS2 (and the related RPS5) are part of a distinct G10 clade (Contreras et al. 2023). The mechanisms by which effector-dependent perturbation of RIN4 activates RPS2, and other CNLs that “guard” RIN4, has remained obscure.

**Figure 7:**
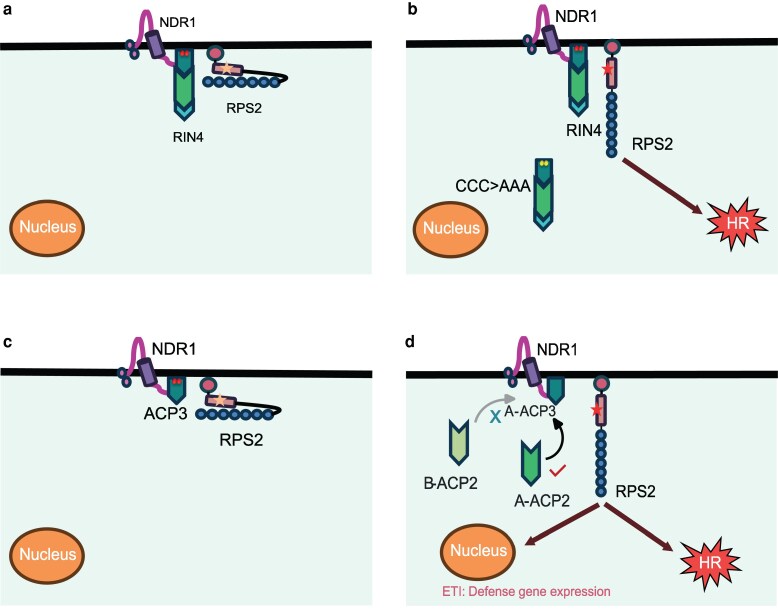
Model of RPS2 resting, pre-activation, and activation complexes. a) In an unstimulated cell, the 2 membrane-tethered NOI domains of RIN4 maintain RPS2 in a resting complex that also includes NDR1. b) A non–membrane-tethered derivative of RIN4 with 2 NOI domains—for example, CCC > AAA—activates RPS2 dependent on NDR1. c and d) The AvrRpt2 cleavage products of RIN4, ACP2, and ACP3, play contrasting roles in RPS2 regulation. (c) ACP3 maintains RPS2 in a pre-activation complex with NDR1. (d) ACP2 interacts with the complex to trigger activation of RPS2. Activation requires both NDR1 and compatibility between ACP2 and ACP3. For example, RPS2 suppressed by ACP3 from plant species “A” is activated by ACP2 from plant species “A” but not by ACP2 from plant species “B”.

Here we demonstrate that non–membrane-tethered derivatives of RIN4 activate RPS2 via a mechanism that is distinct from its ectopic activation in the *absence* of RIN4 (Fig. 7a and b). Furthermore, translating these findings to the activation of RPS2 by AvrRpt2, we demonstrate coordinated roles for ACP2 and ACP3, the AvrRpt2-cleavage fragments of RIN4. While ACP3, along with NDR1, maintains RPS2 within a pre-activation complex (Fig. 7c), NDR1 facilitates interaction of ACP2 with the pre-activation complex to activate RPS2 (Fig. 7d). Triggering of RPS2 in the *presence* of ACP2 is reminiscent of triggering of the apple NLR, Mr5, in the *presence* of ACP3. With the caveat that some findings are based on expression of Arabidopsis proteins in *N. benthamiana*, the data collectively support a model in which cleavage of RIN4 by AvrRpt2 generates both ACP3 with a truncated C-NOI that primes RPS2 and ACP2 that triggers RPS2 activation by interacting with the NDR1-ACP3-RPS2 pre-activation complex. Structural models of interactions between RPS2 and ACP3 or ACP2 align with this model. The structured alpha helix within the C-NOI domain of the otherwise unstructured AtACP3 is predicted with moderate confidence to interact with helical domain 1 of RPS2 (RPS2HD1) (Fig. S10a). Remarkably, the confidence for this predicted interaction between ACP3 from RIN4 of different plant species and RPS2HD1 (Fig. S10b) directly correlates with their ability to suppress RPS2 when coexpressed in *N. benthamiana* (Alam et al. 2021). Furthermore, the alpha helix from within the N-NOI of AtACP2 is also predicted with similar confidence to interact with RPS2HD1 (Fig. S10c) and with an interface that partially overlaps with that of AtACP3 (Fig. S10d).

Our model is also supported by examining regulation of RPS2 in *N. benthamiana* by RIN4 from other angiosperms. RPS2 suppression by ACP3 derivatives from soybean, peach, and potato is relieved only by coexpression of ACP2-like fragments from the same plant species, but not by the comparable fragment from Arabidopsis. Thus, the C- and N-NOI domains of RIN4 appear to have coevolved to maintain functional compatibility, perhaps via their overlapping interactions with RPS2HD1 (Fig. S10d) within the RPS2 activation complex. The idea that the fragments of RIN4 make distinct, cooperative contributions during RPS2 activation is supported by the greater level of similarity within than between C-NOIs and N-NOIs of diverse plant species (Afzal et al. 2011).

Our model is further supported by the observation that activation of RPS2 in Arabidopsis by non–membrane-tethered derivatives of RIN4 requires *NDR1*, which aligns with the requirement of *NDR1* for RPS2 activation by AvrRpt2 and is distinct from the NDR1-independent ectopic activation of RPS2 in *rin4* plants. Surprisingly, AtNDR1 expression in *N. benthamiana* inhibited AvrRpt2-mediated activation of RPS2. A recent study reported that overexpression of NDR1 in Arabidopsis results in interaction of NDR1 with RIN4, which protects it from being cleaved by AvrRpt2 (Samaradivakara et al. 2022). We speculate that overexpression of NDR1 in *N. benthamiana* might suppress RPS2 activation by stabilizing the putative pre-activation complex consisting of RPS2, NDR1, and intact RIN4. Moreover, AtNDR1 expression also suppressed ACP2-mediated activation of ACP3-inhibbited RPS2, suggesting AtNDR1 may have a suppressive function distinct from or additional to preventing cleavage of RIN4.

Inappropriate activation of NLRs in the absence of elicitation is detrimental to plant health. Thus, NLRs must be held in a tightly inactive pre-activation state. Our findings indicate that both NOI domains of membrane-tethered RIN4 contribute to keeping RPS2 inactive, with C-NOI playing a dominant role. While ACP2 can fully activate RPS2 suppressed by ACP3, that has a truncated C-NOI domain, it fails to activate RPS2 suppressed by 142–211, with an intact C-NOI, or RIN4FL, with both NOI domains. The contribution of both NOI domains of membrane-tethered RIN4 in holding RPS2 in an inactive state is mirrored by the contribution of both NOI domains of non–membrane-tethered derivatives in overcoming that suppression. The ability of non–membrane-tethered derivatives of RIN4 with both NOI domains (CCC > AAA or 177Δ211) to overcome the strong suppression of RPS2 by RIN4 is contrasted by the inability of RIN4 derivatives with only a single NOI domain (149Δ211 and ACP2) to do so. Thus, the predominant state of RIN4 in an unchallenged plant, that is, membrane-tethered and containing 2 NOI domains, is likely a key contributor to the minimal activity of RPS2 in the absence of AvrRpt2.

In addition to being tightly suppressed in the absence of activation, NLRs must be specifically and rapidly activated to effectively confer resistance. This can be achieved through formation of pre-assembled complexes responsive to perturbation by an effector. At the plasma membrane, RIN4 associates with both RPS2 and NDR1. While ACP3 can suppress RPS2 in transient assays, it is also part of a pre-activation complex consisting of RPS2, NDR1, and ACP3 (Day et al. 2006; Afzal et al. 2013). Furthermore, the partially truncated C-NOI within ACP3 is essential for triggering of the pre-activation complex by ACP2; ACP2 was unable to activate RPS2 suppressed by a derivative of RIN4 with an intact C-NOI (142–211) or a derivative of ACP3 with a bulky C-terminal adduct. Although ACP2 and other non–membrane-tethered fragments of RIN4 accumulate both in the cytosol and the nucleus, the inability of molecules in the nucleus to activate RPS2 and the required compatibility between ACP2 and ACP3 indicates their likely interaction at the plasma membrane. Thus, when RIN4 is cleaved by AvrRpt2, components previously known (NDR1) and shown here (ACP2 and ACP3) to participate in RPS2 activation are already or immediately present (Fig. S9).

ACP3 suppression of RPS2 contrasts with the regulation of the NLR MR5 in apple, which also responds to AvrRpt2-induced cleave of MdRIN4 but is not ectopically active (Prokchorchik et al. 2020). Instead, AvrRpt2-induced MdACP3 fragment is both necessary and sufficient for MR5 activation, highlighting distinct mechanisms shaped by independent NLR evolution (Prokchorchik et al. 2020). Notably, each model posits that the virulence activity of AvrRpt2, manifested through proteolysis of RIN4, produces the trigger for NLR activation. Recently another independently evolved NLR, Ptr1 from tomato, was demonstrated to respond to AvrRpt2-induced cleavage of SlRIN4 (Mazo-Molina et al. 2020). It will be interesting to determine the role of the AvrRpt2-induced fragments of SlRIN4 in the activation of Ptr1.

AvrRpt2 promotes *P. syringae* growth in plants lacking RIN4, indicating that targets additional to RIN4 can contribute to its virulence activity (Lim and Kunkel 2004). Candidates for these virulence targets include a family of Arabidopsis proteins containing a single NOI domain, which are known to be proteolytically targeted by AvrRpt2 (Chisholm et al. 2005; Eschen-Lippold et al. 2016). Given that these “single-NOI” proteins also share a similar C-terminal acylation motif with RIN4, their cleaved products may share a similar activity with ACP3, which we have shown is a potent suppressor of Arabidopsis defense (Afzal et al. 2011). Non-membrane-tethered ACP2 is also a potent suppressor of Arabidopsis defense but, unlike ACP3, it is also able to suppress flg22-induced callose deposition (Afzal et al. 2011). Whether the nuclear localization of ACP2 contributes to its ability to suppress plant immunity remains unclear. In either case, AvrRpt2 may make a quantitatively stronger and distinct contributions to bacterial virulence by targeting RIN4, relative to single-NOI proteins. Thus, RPS2 may have evolved to “guard” RIN4, rather than a single-NOI protein, because its mechanism for perception of AvrRpt2 depends on production of the locally concentrated, but non-membrane-tethered, NOI cleavage product, ACP2 (Fig. S9). This model is consistent with a prediction, based on the guard hypothesis, that RPS2 is triggered by the virulence-promoting perturbation produced by AvrRpt2.

## Methods

### Experimental models and details

#### *Arabidopsis thaliana* and *nicotiana benthamiana*

*Arabidopsis thaliana* (Col-0, transgenic and mutants) and *Nicotiana benthamiana* plants were grown in potting soil under a cycle of 8 h light at 23 °C and 16 h dark at 16 °C.

#### Escherichia coli

*E. coli* (Dh5 or Top10) carrying the respective constructs were grown at 37 °C in Lb medium containing the appropriate antibiotic.

#### Agrobacterium tumefaciens

*Agrobacterium tumefaciens* (strain GV-3101) carrying expression constructs or empty vectors were grown overnight at 28 °C in LB medium containing appropriate antibiotics (Rifampicin, Gentamicin and Kanamycin, Spectinomycin or Tetracycline).

## Method details

### Constructs

All constructs for RIN4 derivatives (Fig. 1a) were derived from pMAC100c vector containing full-length RIN4 coding sequence (Afzal et al. 2011). These derivatives include the CCC > AAA (mutation of acylation site cysteines to alanines), 177Δ211 (deletion of 35 C-terminal residues of RIN4), 11–211 (Deletion of ACP1), ACP2 (11–152), ACP3 (153–211), 142–211 (similar to ACP3, but contains the full C-NOI), ΔΔNOI (deletion of 1Δ64 and 149Δ176), 203Δ211 (deletion of 8 C-terminal residues of RIN4), and 149Δ211 (deletion of 62 C-terminal residues of RIN4). The RIN4 derivatives were fused with an N-terminal epitope tag [T7 (MASMTGGQQMG), ACV5 (SWKDASGWS), or Flag (DYKDDDDK)] or with yellow fluorescent protein (YFP)]. For localization studies, Nuclear Localization Signal (NLS, PKKKRKVED) (Haasen et al. 1999), Nuclear Export Signal (NES, NELALKLAGLDINKT) (Gadal et al. 2001), or Shuffled Nuclear Export signal (SNE, NELALKAAGADINKT) tags were added between the N-terminal florescent/epitope tags and the C-terminal sequences encoding RIN4 or its derivatives. For transient studies in *N. benthamiana*, these chimeric fragments were cloned into pENTR-D-TOPO and subsequently moved into the gateway binary vectors pB2GW7or pGWB12 (containing a 35S promoter) or pEarlyGate 104 (35S:N-YFP).

Both 35S promoter driven (35S:) and estradiol inducible (Est:) RPS2:Turbo-ID:V5 and BRI1:TurboID constructs were generated through golden gate cloning (Werner et al. 2012). YFP:RIN4FL, YFP:AcV5:INT (internal) and YFP:ACP3 expressed under the control of a 35S promoter (pEarley gate 104 vector backbone) have been described previously (Alam et al. 2021). RPS2:HA, expressed under the control of a strong promoter (in pOCS), and HA:NDR1 have been described previously (Day et al. 2005, 2006). 35S:AvrRpt2:HA and 35S:AvrRpt2^C122A^:HA have been described previously (Prokchorchik et al. 2020). 35S:RFP:OsRac1 (pGDR vector backbone), used as a plasma membrane marker, has been described previously (Fan et al. 2018).

### Plant transformations

Transgenic Arabidopsis lines expressing dexamethasone (dex)-inducible tagged derivatives of RIN4 were generated as previously described (Afzal et al. 2011) in Col-0, *ndr1-1*, *rpm1-3*, *rps2-101c*, or *rpm1rps2* backgrounds. Transgene expression was induced by painting single leaves of T1 plants or spraying T2 and T3 plants with a solution containing 20 μM dex (Sigma-Aldrich, St. Louis, MO, USA) and 0.05% Silwet L-77 (Momentive, Waterford, NY, USA). T1 families showing a segregation ratio of 3:1 for resistance:susceptibility to hygromycin were self-fertilized and propagated to homozygosity.

### Transient expression assays

*Agrobacterium tumefaciens* (strain GV-3101) carrying expression constructs or empty vectors were grown overnight at 28 °C in LB medium containing appropriate antibiotics (rifampicin, gentamicin and kanamycin, spectinomycin or tetracycline). Cultures were centrifuged at 4,000 rpm for 20 min and the cell pellets were subsequently re-suspended in infiltration buffer (10 mM MgCl_2_, 10 mM MES, and 200μM Acetosyringone adjusted to pH 5.6 with KOH). For all infiltration experiments the OD_600_ of cultures was adjusted with infiltration buffer and when necessary, the final OD_600_ was held constant by supplementing with bacteria carrying an empty vector. Final cultures were infiltrated into *N. benthamiana* leaves as described previously (Tai et al. 1999; Day et al. 2005).

### Immunoblot analysis

Immunoblot analysis was performed as described previously (Afzal et al. 2011). Briefly, plant material was homogenized in a buffer containing 20 mM Tris-HCl [pH 7.5], 150 mM NaCl, 1 mM EDTA, 1% Triton X-100, 0.1% SDS, 5 mM DTT, and 1 × plant protease inhibitor cocktail and centrifuged at 20,000 × g for 10 minutes at 4 °C. The concentration of protein in the supernatant was determined by the Bio-Rad protein assay reagent (Bio-Rad, Hercules, CA, USA) according to manufacturer's instructions. Anti-RIN4 sera (Mackey et al. 2002), anti-T7 monoclonal antibody (Novagen, Madison, WI, USA), anti-AcV5 antibody (eBioscience, San Diego, CA, USA), anti-Flag antibody (Sigma-Aldrich, Taufkirchen, Germany) and anti-YFP antibody (Abcam, Cambridge, UK) were used at dilutions of 1:5,000, 1:10,000, 1:5,000, 1:5,000, or 1:5,000, respectively. Chemiluminescent detection and band quantification were done using the ChemiDoc XRS system (Bio-Rad) and ImageJ (imagej.nih.gov/ij/).

### TurboID-based immunoprecipitation (IP)

For TurboID-IP assays with either 35S:RPS2:TurboID:V5 or 35S:BRI1:TurboID:V5 and additional constructs were transiently expressed in 4-wk-old *N. benthamiana* leaves. After 24 h, the infiltrated leaves were treated with 50 µM biotin for 2 h. The leaves were then ground to a fine powder in liquid nitrogen and homogenized in extraction buffer (Glycerol 10%, 150 mM Tris-HCl [pH7.5], 1 mM EDTA and 150 mM NaCl, 10 mM DTT, 0.4% Nonidet-40 (Igepal), Anti-protease cocktail, 2% PVPP and 0.5% sodium deoxycholate (w/v)). The supernatant from homogenized samples was centrifuged 3 times at 5,000 g at 4 °C for 10 min. To remove cell wall debris the supernatant was passed through miracloth into Zeba Spin Desalting Columns (Thermo Fisher Scientific, Catalog number 89,893) and subsequently centrifuged for 1 min at 4 °C to remove excess biotin. A total of 120 µL of flowthrough was collected as input, while the rest was incubated at 4 °C with 30 µL streptavidin-coated beads (Pierce High Capacity Streptavidin Agarose, Thermo Fisher, Catalogue number 20,361). After 2 h, the beads were spun down at 5,000 g at 4 °C for 3 min and washed 4 times with washing buffer (glycerol 10%, 150 mM Tris-HCl [pH7.5], 1 mM EDTA and 150 mM NaCl, 10 mM DTT, 0.4% Nonidet-40 (Igepal), Anti-protease cocktail and 0.5% sodium deoxycholate (w/v)). Finally, 3 × SDS loading buffer (3.3% SDS, 94 mM Tris-HCl pH 6.8, 30% glycerol and 0.05% (vol/vol) bromophenol blue) were added to beads and input samples followed by boiling at 95 °C for 7 min to denature proteins. Denatured proteins were analyzed by immunoblots.

TurboID-IP assays with Est:RPS2:TurboID:V5 or Est:BRI1:TurboID:V5 were done as described above. However, the leaves were treated with 50 µM biotin and 50 nM estradiol 48 h post agroinfiltration. The samples were harvested 3 h after biotin and estradiol treatment.

### Measurement of electrolyte leakage

For measurement of ion leakage, Arabidopsis leaf discs (6–8 mm diameter) were collected 48 h post dex treatment and were immersed in a 50-mL sterile tube containing 15 mL of water. For transient assays in *N. benthamiana*, leaf discs (9–10 mm) collected from the agro-infiltrated region at either 48 or 72 HPI were immersed in a 50 mL sterile tube containing 15 mL of water. Conductance was measured at the indicated time points using a conductivity meter (WTW, Weilheim, Germany). In both cases, leaf discs from non-infiltrated leaves were also collected to establish background ion leakage. Each assay included 3 to 5 separate biological replicates, with 2 to 3 technical replicates measured at a given time point. For data presented as percent ion leakage, samples were measured then boiled and remeasured to determine maximum ion leakage.

### Confocal microscopy

*N. benthamiana* leaves were infiltrated with Agro carrying the indicated construct(s). At 48 or 72 h after infiltration, YFP and/or RFP florescence were observed with a confocal microscope (Nikon Eclipse Ti/C2/C2Si) (Nikon, Foster City, CA, USA) using an excitation wavelength of 514 nm and emission wavelength of 530 nm or an excitation wavelength of 561 nm and an emission wavelength of 575 nm, respectively. Nuclei were stained by infiltrating 0.4 ug/mL Hoechst 33342 dye (Sigma-Aldrich, St. Louis, MO, USA) into leaves 8 h before image acquisition (Fang and Spector 2010) and observed using an excitation wavelength of 350 nm and an emission wavelength of 460 nm.

### Trypan blue staining

Trypan blue staining was performed as described (Afzal et al. 2011). Briefly, staining solution was prepared by mixing 1 part staining mix (1:1:1:1 mix of phenol, lactic acid, glycerin, and water plus 0.05% [w/v] trypan blue) and 2 parts ethanol. The leaves were submerged in the staining solution for 3 min at 95 °C followed by an additional overnight incubation. Stain was removed by 15 M chloral hydrate solution and mounted in 70% glycerol.

### AlphaFold predictions

Protein complexes were predicted using AlphaFold3 (Abramson et al. 2024) with default parameters and visualized in ChimeraX (Meng et al. 2023). Solid lines between chains indicate residue pairs closer than 4 Angstroms and are colored according to the AlphaFold predicted aligned error (PAE), as implemented in the AlphaFold Contacts tool in ChimeraX.

### Statistical analysis

Analyses were conducted as described in the text and figure legends. Statistical data are provided in Data Set S1.

### Accession numbers

AvrRpt2 (GenBank: L11355.1); RIN4 (AT3G25070); NDR1 (AT3G20600); RPS2 (AT4G26090).

## Supplementary Material

koag204_Supplementary_Data

## Data Availability

All data associated with the study are in the paper or supplemental information and can also be requested from the lead contact.
