# Supplementary material for: RIN4 fragments released by AvrRpt2 promote NDR1-dependent activation of RPS2
Source: Plant Cell. 2026 Jul 7;38(7):koag204. doi: 10.1093/plcell/koag204 (PMC13378167; doi:10.1093/plcell/koag204)
Supplement: koag204_Supplementary_Data [file koag204_supplementary_data.zip › Alam et al supplement 06232026.pdf]

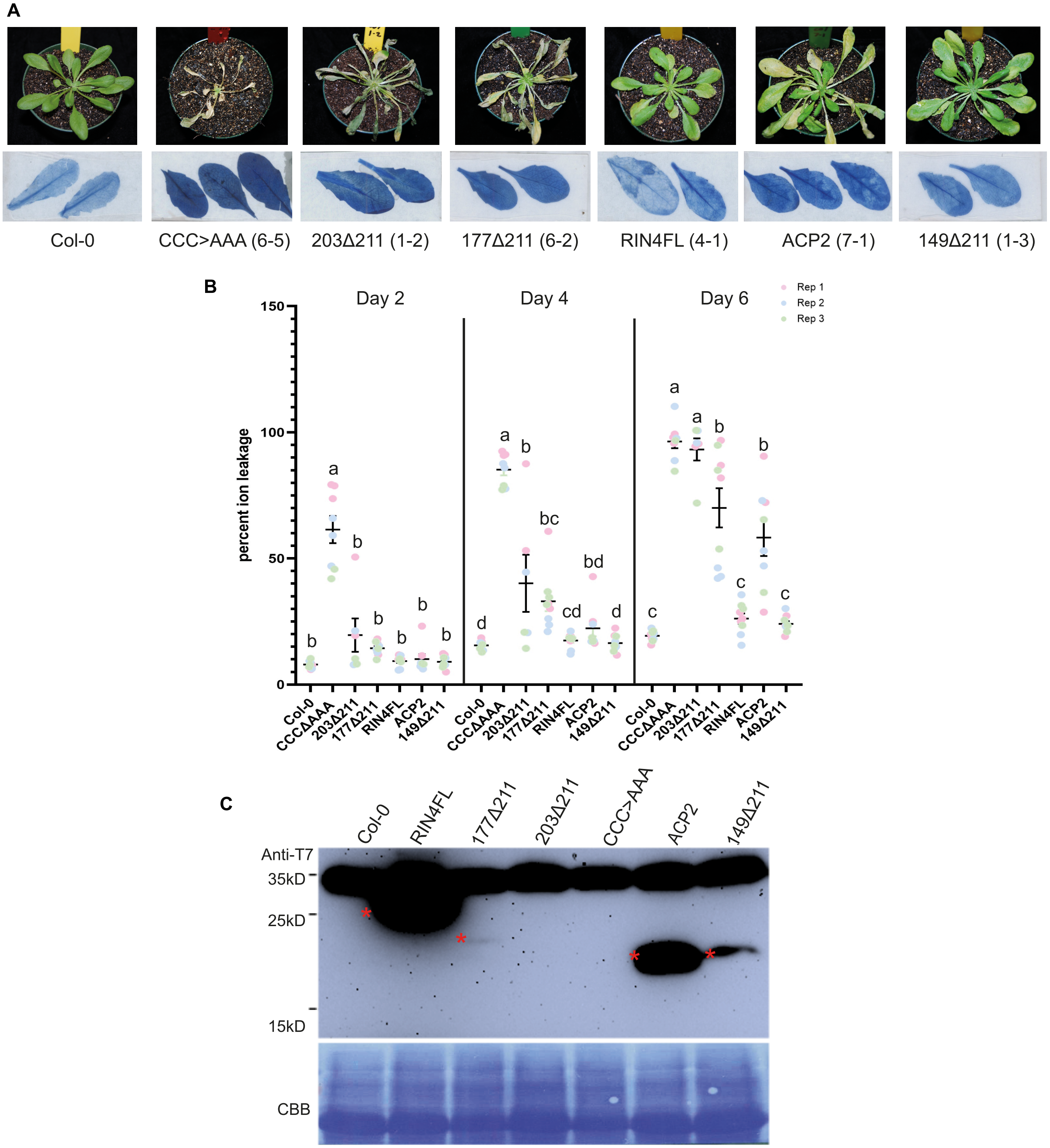

Supplementary Figure S1: Membrane-tethered derivatives of RIN4 that contain an NOI domain cause chlorosis, but not cell death (Supports Figure 1). **A)** Expression of RIN4 derivatives in homozygous transgenic Arabidopsis plants was induced by dex. Macroscopic symptoms were observed at 72 HPI. **Bottom panel** Trypan blue staining of leaves from plants as in A. **B)** Cell death in plants as in A was quantified by measuring electrolyte leakage. Data was collected from 3 independent experiments with at least 2-3 technical replicates per transgenic line (n=3-9). Error bars represent SEM. Statistical analysis was performed using one-way ANOVA, followed by Tukey's multiple comparison test (Refer to supplementary data set 1). **C)** Anti-T7 western blot shows accumulation of RIN4 derivatives at 72 HPI in the indicated transgenic lines. The lower panel shows Coommasie brilliant blue stain for RuBisCO used as loading control. Asterisks indicate the positions of individual RIN4 derivatives.

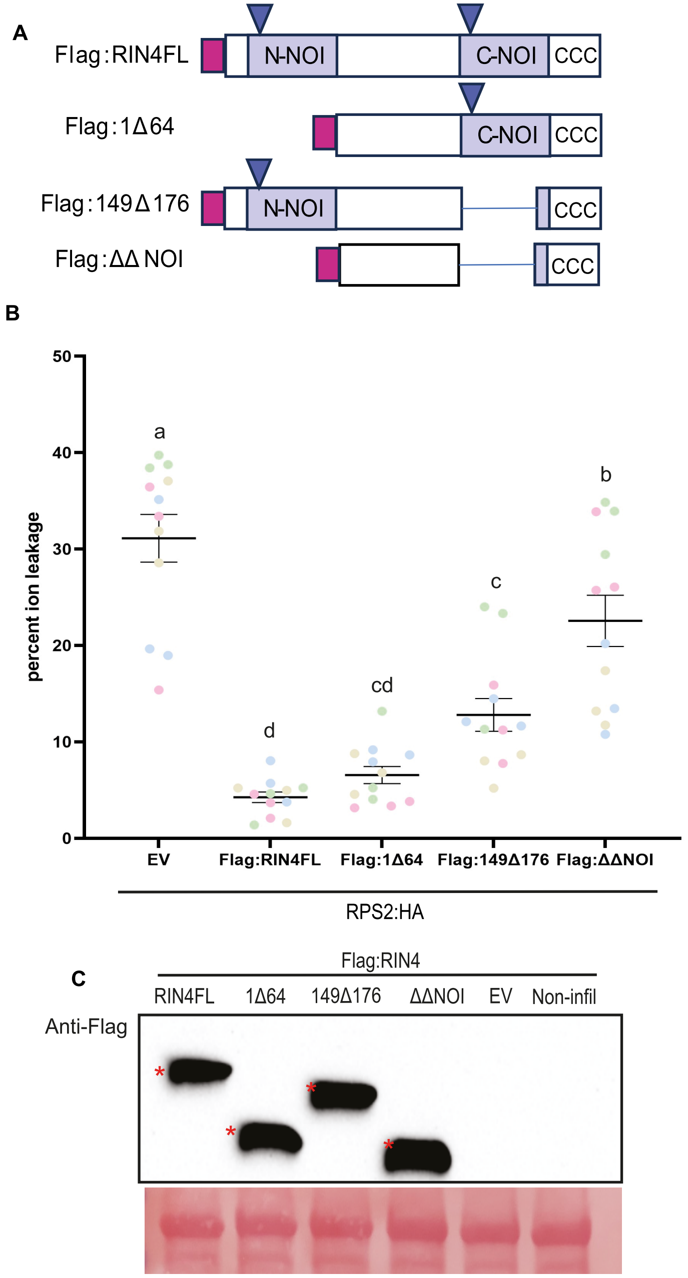

Supplementary Figure S2: Suppression of RPS2 in *N. benthamiana* by membrane-tethered derivatives of RIN4 depends on C-NOI and, to a lesser extent, N-NOI (Supports Figure 1). **A)** Flag:RIN4 derivatives expressed from 35S promoter in transient experiments in *N. benthamiana*. **B)** The indicated derivatives of RIN4 (OD_{600} = 1.0) and RPS2-HA (OD_{600} = 0.05) were co-infiltrated in *N. benthamiana*. Cell death in plants was quantified by measuring electrolyte leakage at 96 HAI. Data was collected from 3 independent experiments with 3 technical replicates per treatment (n=9). Error bars represent SEM. Statistical analysis was performed using one-way ANOVA, followed Tukey's multiple comparison test (Refer to supplementary data set 1). **C)** Anti-Flag immunoblot shows accumulation of Flag:RIN4 derivatives expressed in *N. benthamiana* at 72 HAI. The lower panel shows ponceau stain for RuBisCO used as loading control. Asterisks indicate the positions of individual RIN4 derivatives.

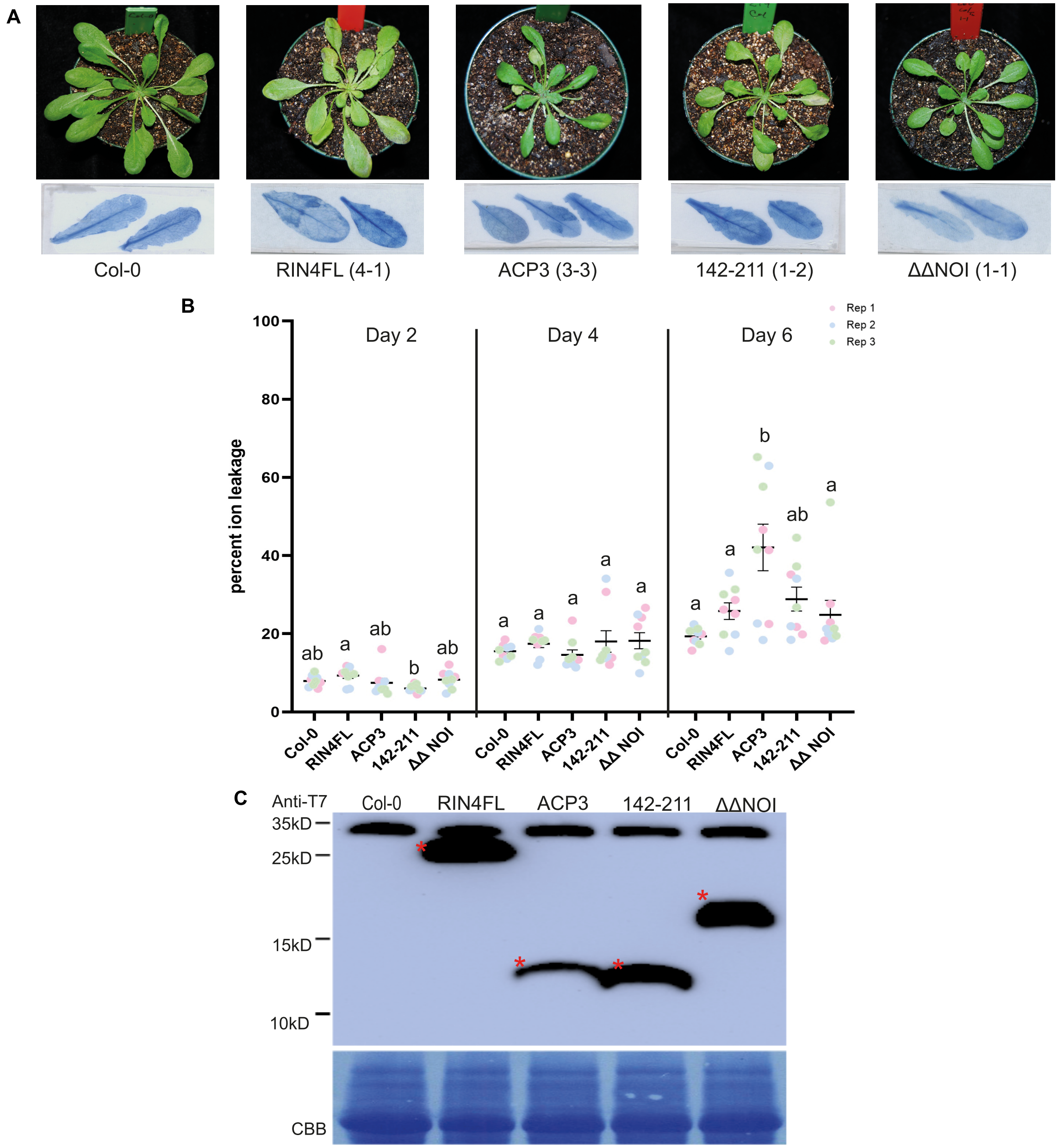

Supplementary Figure S3: Non-membrane-tethered derivatives of RIN4 depend on their C-NOI domain to cause a cell death response in Col-0 (Supports Figure 1). **A)** Expression of RIN4 derivatives was induced by dex treatment in homozygous transgenic Arabidopsis plants. Macroscopic symptoms were observed at 72 HPI. **Lower panel:** Trypan blue staining of leaves from plants as in A. Images for Col-0 and RIN4FL are the same as shown in FigS1A.**B)** Cell death in plants as in A was quantified by measuring electrolyte leakage. Data was collected from 3 independent experiments with at least 3 technical replicates per transgenic line (n=3-9). Error bars represent SEM. Statistical analysis was performed using one-way ANOVA, followed by Tukey's multiple comparison test (Refer to supplementary data set 1). **C)** Anti-T7 immunoblot showing protein accumulation of RIN4 derivatives in the indicated transgenic lines at 72 HPI. The lower panel shows coommasie brilliant blue stain of RuBisCO as a loading control. Asterisks indicate the positions of individual RIN4 derivatives.

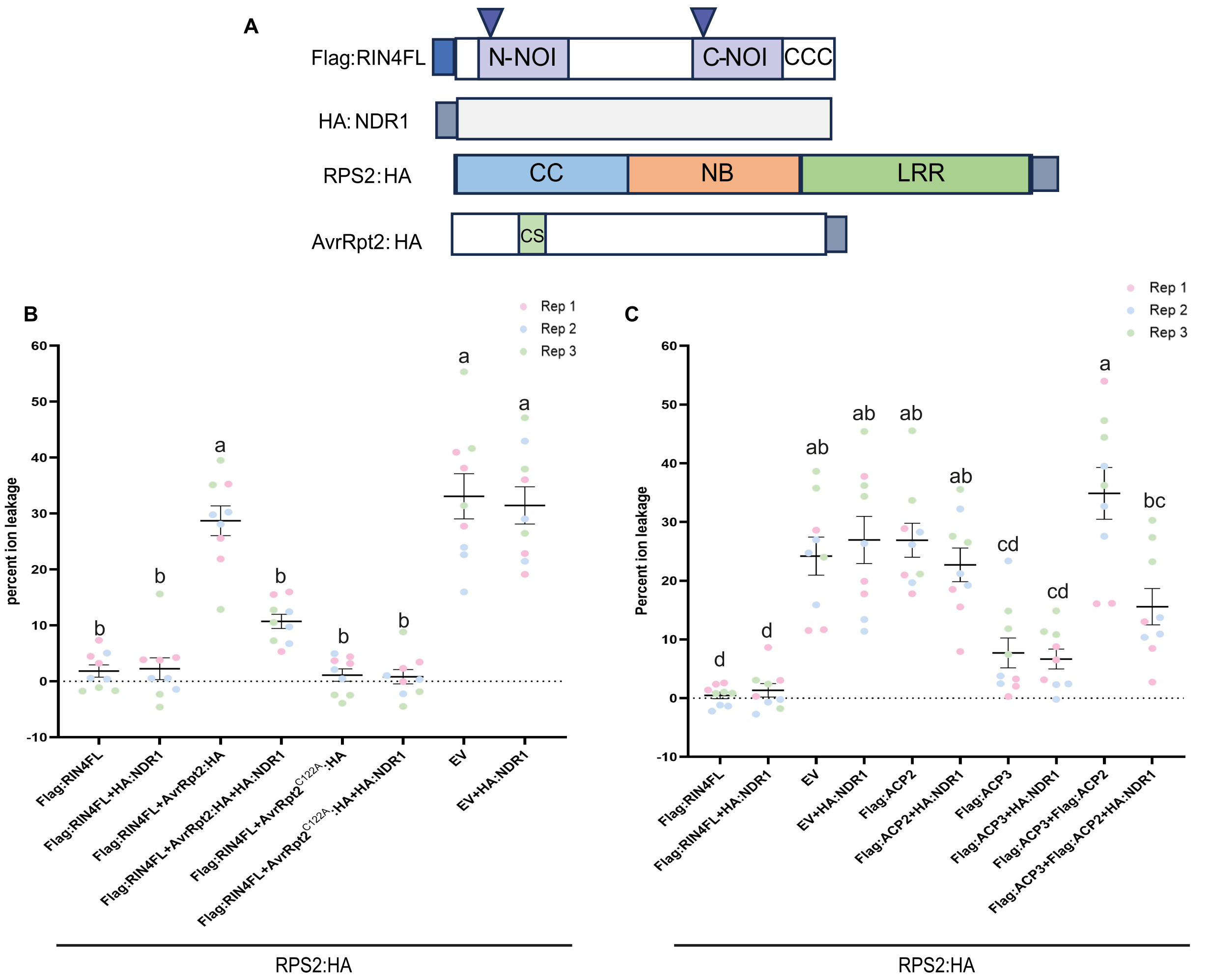

Supplementary Figure S4: Overexpression of AtNDR1 in *N. benthamiana* suppresses RPS2-induced cell death (Supports Figure 2 and 4). **A)** Flag:RIN4Fl, RPS2:HA, HA:NDR1 and AvrRpt2:HA constructs used in transient experiments in *N. benthamiana*. **B)** The indicated combinations of Flag:RIN4Fl (OD600 0.6), HA:NDR1 (OD_{600} = 0.6), AvrRpt2:HA and/or AvrC122A:HA (OD_{600} = 0.01) and RPS2:HA (OD_{600} = 0.08), were co-infiltrated in *N. benthamiana*. Cell death in plants was quantified by measuring electrolyte leakage at 72 HAI. Error bars represent SEM. Data was collected from 3 independent experiments with 3 technical replicates per treatment (n=9). **C)** The indicated combinations of RIN4 derivatives (OD_{600} 0.6), with HA:NDR1 (OD_{600} = 0.6) and RPS2:HA (OD_{600} = 0.05), were co-infiltrated in *N. benthamiana*. Cell death in plants was quantified by measuring electrolyte leakage at 96 HAI. Error bars represent SEM. Data was collected from 3 independent experiments with 3 technical replicates per treatment (n=12). B-C) Statistical analysis was performed using one-way ANOVA, followed by Tukey's multiple comparison test (Refer to supplementary data set 1).

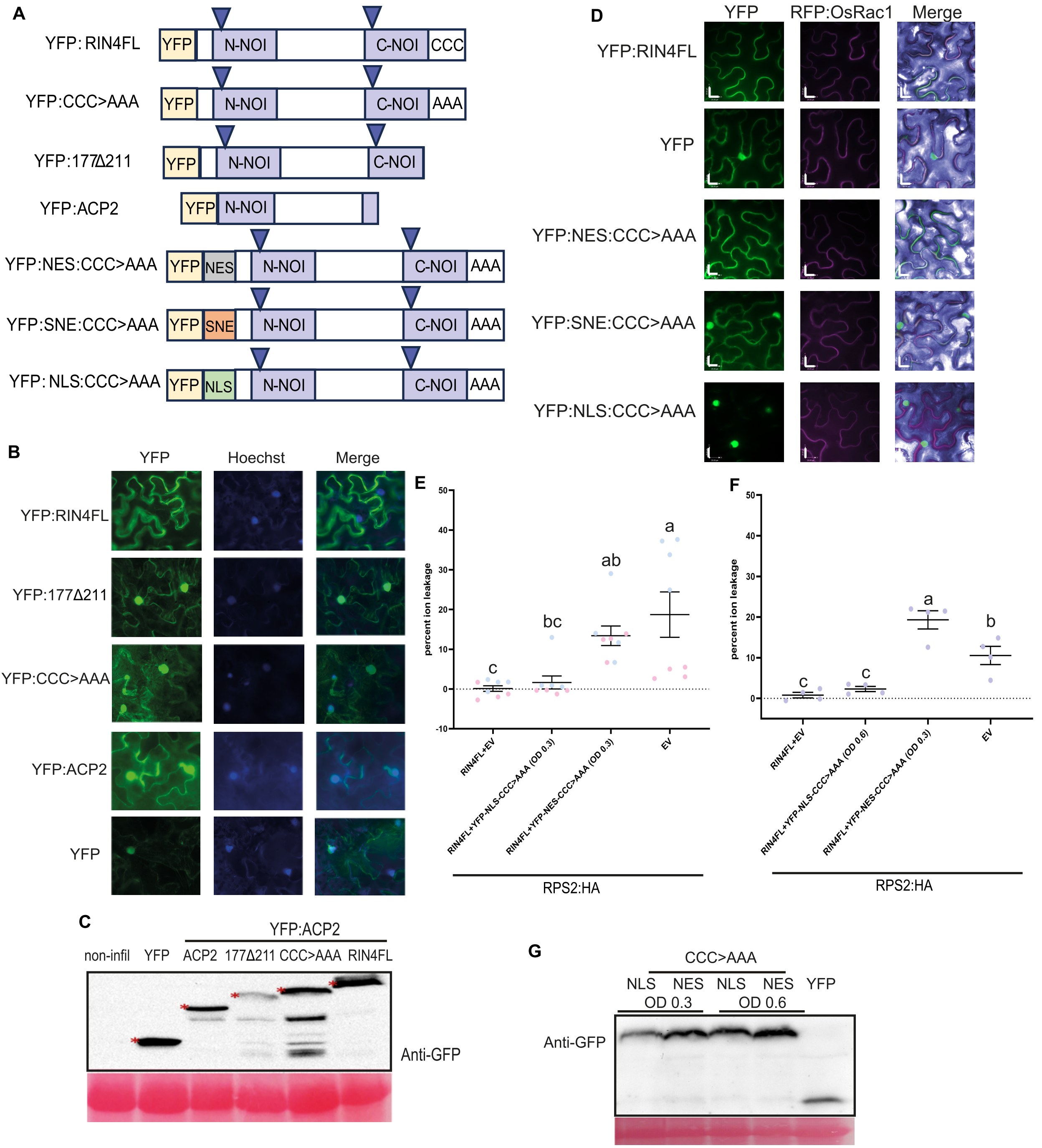

Supplementary Figure S5. Activation of RPS2 in *N. benthamiana* by non-membrane-tethered RIN4 requires its accumulation in the cytosol (Supports Figure 3). **A)** YFP taggedRIN4 derivatives expressed from 35S promoter in transient experiments in *N. benthamiana*. N-terminal rectangles, NLS (green), NES (grey), or SNE (orange) tags. **B)** The indicated RIN4 derivatives were expressed in *N. benthamiana*. Shown are YFP, Hoechst, and merged confocal microscope images at 48 HAI and following Hoechst staining. **C)** Anti-GFP immunoblot shows accumulation of RIN4 derivatives expressed in *N. benthamiana*. The lower panel shows

ponceau staining for RuBisCO, used as loading control. Asterisks indicate the positions of individual RIN4 derivatives. **D)** The indicated RIN4 derivatives or free YFP were co-expressed with RFP-OsRac1 in *N. benthamiana*. Shown are YFP, RFP, and merged confocal microscope images at 72 HPI. Scale bars are 15 μM. **E-F)** The indicated combinations of Flag-RIN4Fl (OD_{600} = 1.0), either YFP-NLS-CCC>AAA (OD_{600} = 0.6 or 0.3) or YFP-NES-CCC>AAA (OD_{600} = 0.3) and RPS2-HA (OD_{600} = 0.07), were co-infiltrated in *N. benthamiana*. Cell death in plants was quantified by measuring electrolyte leakage at 48 HAI. Error bars represent SEM. Statistical analysis was performed using one-way ANOVA, followed by Tukey's multiple comparison test (Refer to supplementary data set 1). G) Anti-YFP western blot shows expression levels of YFP-NLS-CCC>AAA and YFP-NES-CCC>AAA at 48 HPI. Panels below show ponceau stain for RuBisCO used as loading control. Arrow indicates positions of the RIN4 derivatives.

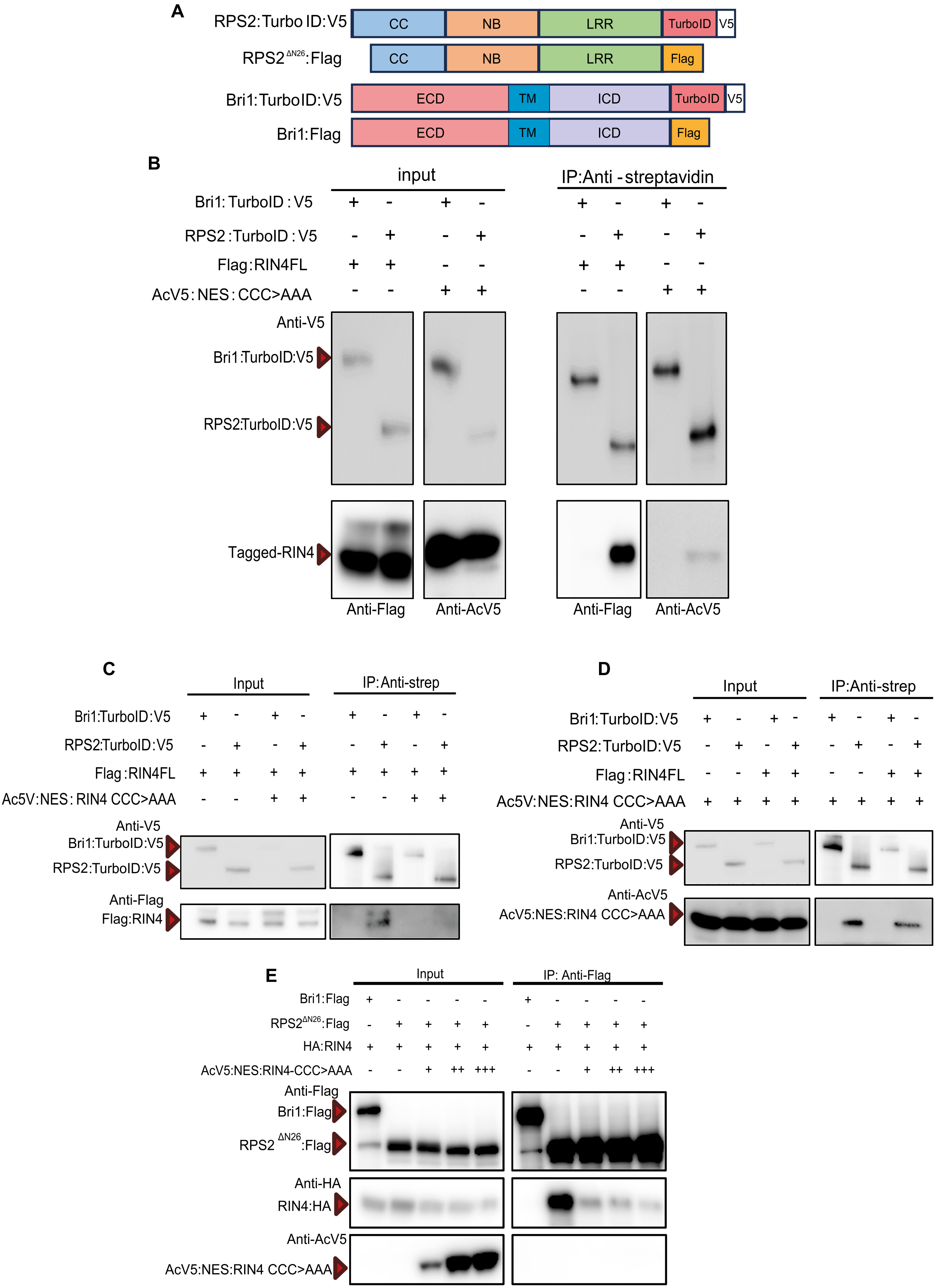

A
RPS2:Turbo ID:V5
CC
NB
LRR
TurboID
V5
RPS2 ΔN26:Flag
Flag
Bri1:TurboID:V5
ECD
TM
ICD
Bri1:Flag
B
input
IP:Anti -streptavidin
Bri1: TurboID : V5
RPS2 :TurboID : V5
Flag:RIN4FL
AcV5: NES: CCC>AAA
Anti-V5
Bri1:TurboID:V5
RPS2:TurboID:V5
Tagged-RIN4
Anti-Flag
Anti-AcV5
C
Input
IP:Anti-strep
Bri1:TurboID:V5
RPS2:TurboID:V5
Flag:RIN4FL
Ac5V:NES: RIN4 CCC>AAA
Anti-V5
Anti-Flag
Flag:RIN4
D
Input
IP:Anti-strep
Bri1:TurboID:V5
RPS2:TurboID:V5
Flag:RIN4FL
Ac5V:NES:RIN4 CCC>AAA
Anti-V5
Anti-AcV5
AcV5:NES:RIN4 CCC>AAA
E
Input
IP: Anti-Flag
Bri1:Flag
RPS2ΔN26:Flag
HA:RIN4
AcV5:NES:RIN4-CCC>AAA
Anti-Flag
Bri1:Flag
RPS2 ΔN26:Flag
Anti-HA
RIN4:HA
Anti-AcV5
AcV5:NES:RIN4 CCC>AAA

Supplementary Figure S6: RPS2:TurboID:V5 specifically biotinylates Flag:RIN4 and AcV5:NES:RIN4 (Supports Figure 3). **A)** tagged 35S:RPS2, 35S:RPS2$^{\Delta N26}$ and 35S:Bri1 constructs. **B)** RIN4 derivatives and either RPS2:TurboID:AcV5 or Bri1:TurboID:V5 (each at OD_{600} = 0.5) were co-infiltrated in *N. benthamiana* leaves. After 24hrs the leaves were infiltrated with biotin and 4hrs later the tissue was collected. A portion was immunoprecipitated with anti-strep and the input and IP samples were immunoblotted as indicated with red arrows indicating the position of the proteins. **C-D)** RIN4FL, NES:RIN4-CCC>AAA and either RPS2:TurboID:V5 or Bri1:TurboID:V5 were co-infiltrated in *N. benthamiana* leaves (each at OD_{600} = 0.5) and were processed as in B. V5-HRP was used to detect RPS2 and Bri1, while Flag-HRP and anti-AcV5 was used for detection of biotinylated proteins (bottom panels). **E)** HA:RIN4FL, AcV5:NES:RIN4-CCC>AAA and either RPS2$^{\Delta N26}$:Flag or Bri1:Flag were co-infiltrated in *N. benthamiana* leaves (each at OD_{600} = 0.5). Flag-HRP was used to detect RPS2 and Bri1, while AcV5-HRP and HA-HRP was used for detection of RIN4 derrivatives.

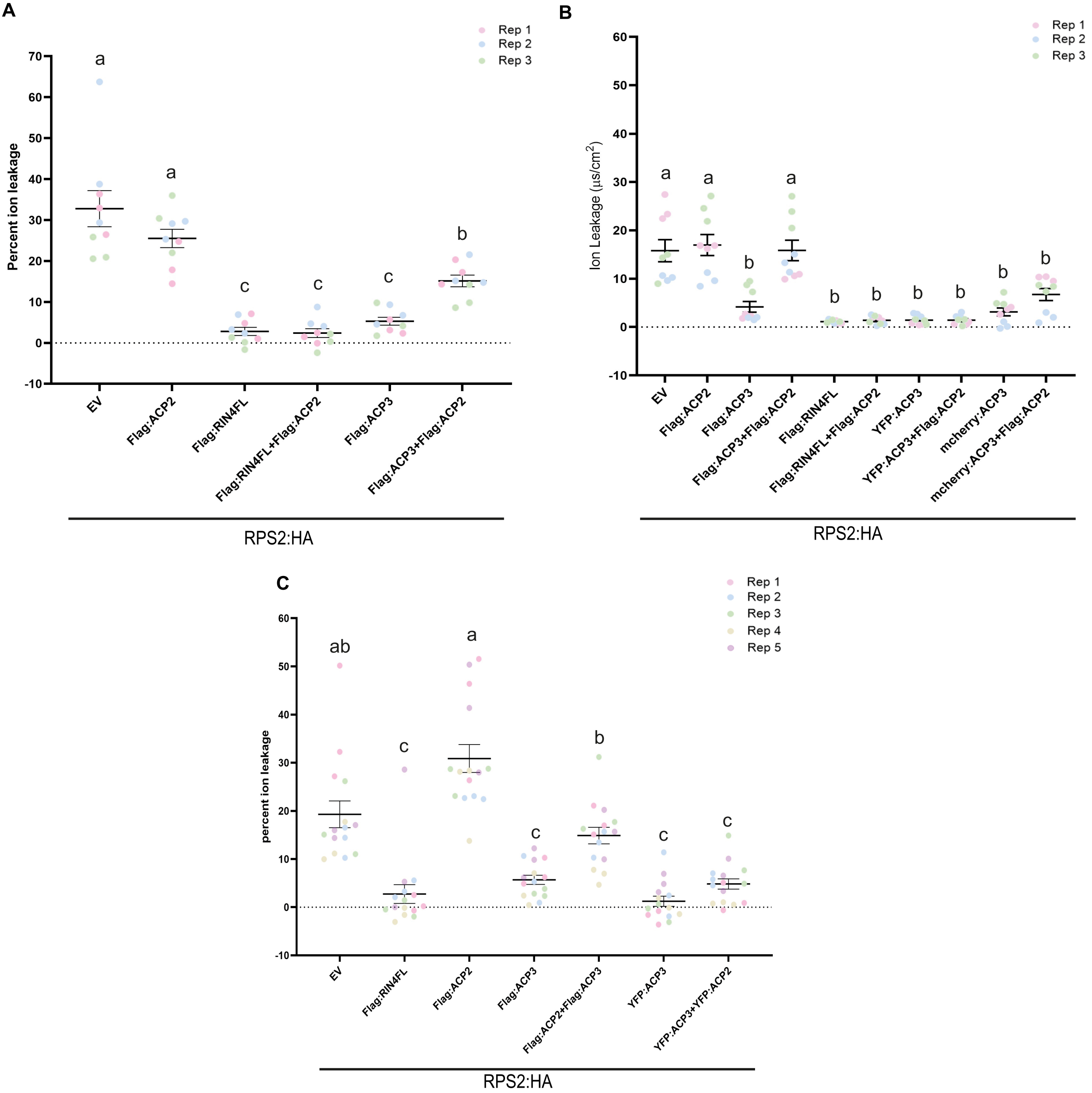

Supplementary Figure S7. ACP2 is unable to activate RPS2 suppressed by full-length RIN4 or ACP3 with a bulky N-terminal tag in *N. benthamiana* (Supports Figure 4 and 5). **A-C)** The indicated RIN4 derivatives (OD_{600} = 0.6) and RPS2-HA (OD_{600} = 0.04) were co-infiltrated in *N. benthamiana*. Cell death in plants was quantified based on electrolyte eakage. Leaf discs were immersed in sterile water and conductivity was measured at 96 HAI. **A and B)** Data was collected from 3 independent experiments with 3 technical replicates per treatment (n=9). Error bars represent SEM. **C)** Data was collected from 5 independent experiments with 3 technical replicates per treatment (n=15). Error bars represent SEM. Statistical analysis was performed using one-way ANOVA, followed by Tukey's multiple comparison test (Refer to supplementary data set 1).

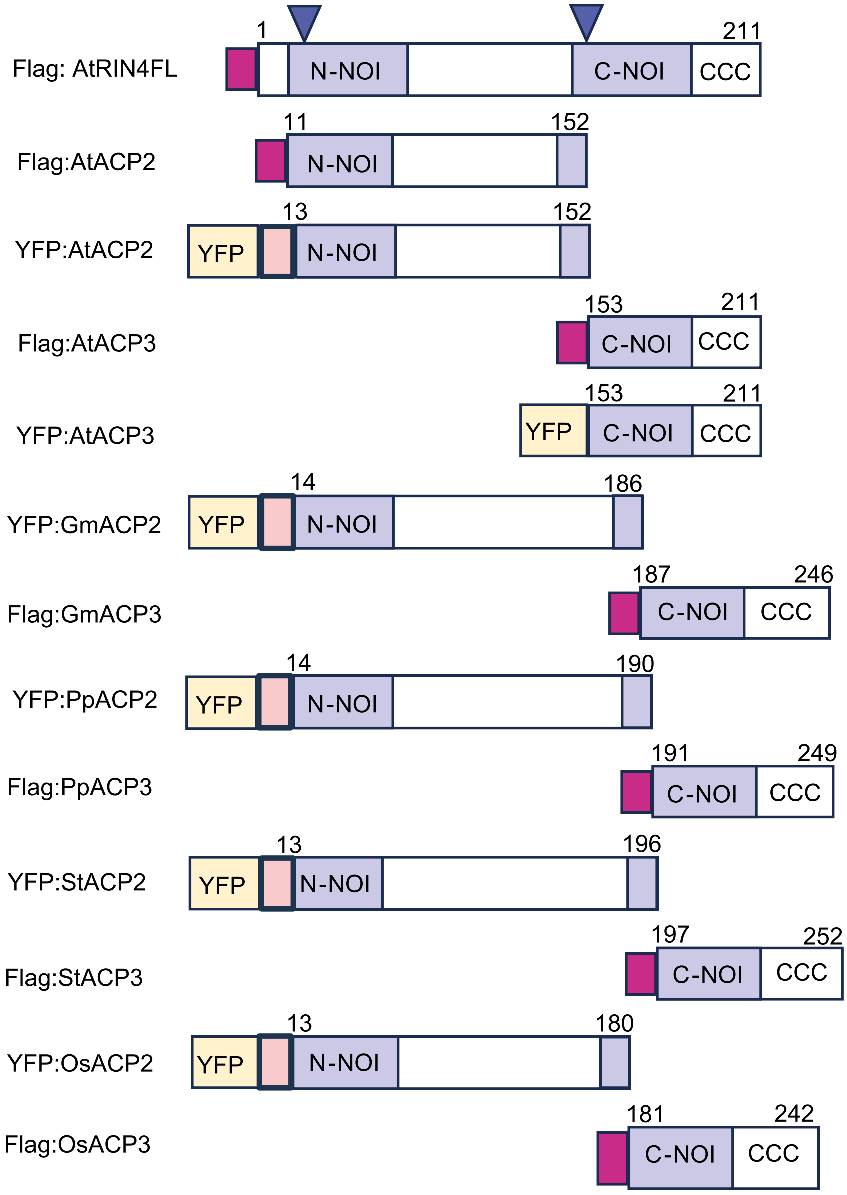

Supplementary Figure S8: Schematic diagram of homologous RIN4 derivatives (Supports Figure 6). Within RIN4 derivatives: Yellow rectangles indicate YFP tag, peach rectangle indicates AcV5 epitopes, pink triangle indicates Flag epitope, purple rectangles indicate NOI domains, and blue triangles indicate RCS.

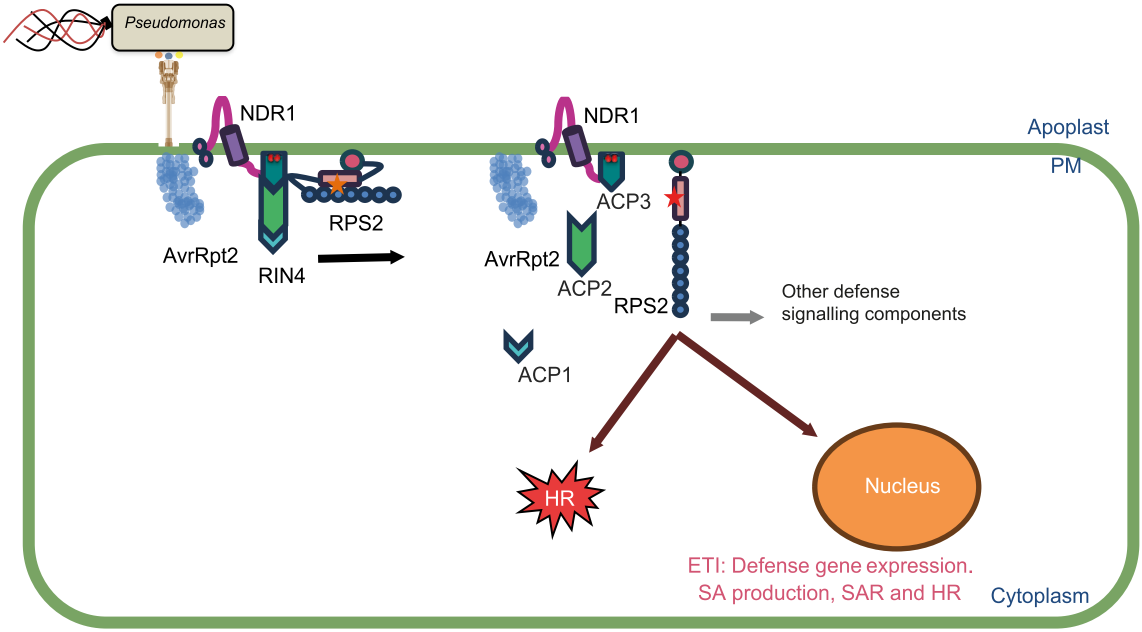

Supplementary Figure S9: The schematic diagram of AvrRpt2 induced activation of RPS2 in Arabidopsis (Supports Figure 7). In the absence of the pathogen, membrane localized RIN4 suppresses RPS2 activation. *P. syringae* secretes AvrRpt2, a cysteine protease, in plant cytosol using the T3SS. AvrRpt2 cleaves RIN4 at the conserved motif VPXFGXW, known as RCS1 and RCS2 (RIN4 cleavage sites), in the N and the C-NOI domains respectively. Cleavage of RIN4 at these sites results in the generation of three fragments, namely ACP1, ACP2 and ACP3. The production of non-membrane tethered fragment, ACP2, elicits RPS2 activation in the presence of the membrane tethered fragment, ACP3. Activated RPS2 in turn triggers defense gene expression and HR.

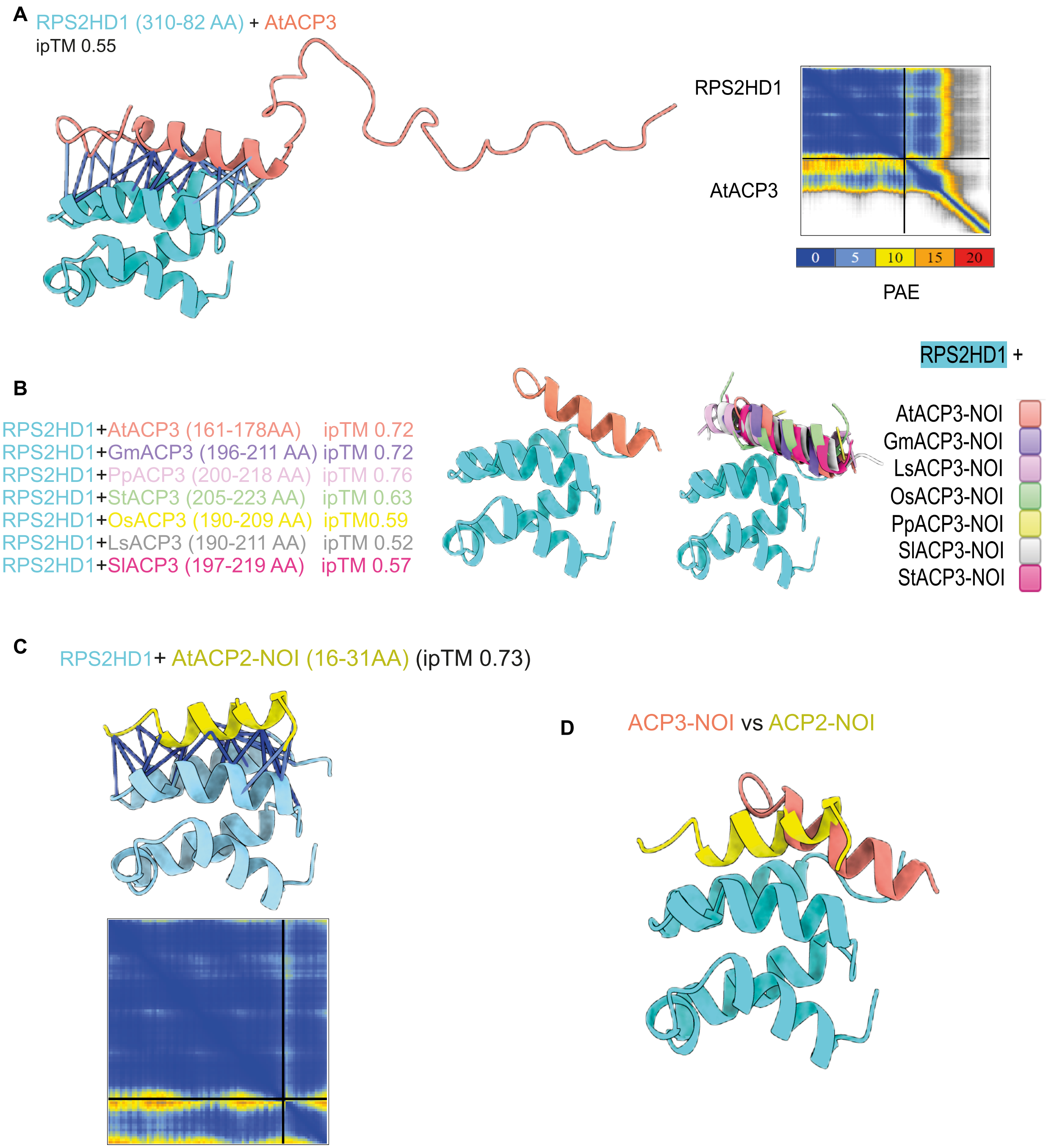

Supplementary Figure 10: Structural modeling of interactions between RIN4 and RPS2 (Suppprts Figure 4, 6 and 7). **A)** AlphaFold3 prediction of the interaction between RPS2HD1 and AtACP3. Solid lines indicate residue–residue contacts between the two chains within 4 Å and are coloured according to predicted aligned error (PAE) values. **B)** AlphaFold3-predicted interaction between RPS2HD1 and ACP3-NOI from different species. **C)** AlphaFold3 prediction of the interaction between RPS2HD1 and AtACP2-NOI. **D)** Comparison of ACP2-NOI and ACP3-NOI binding to RPS2HD1. Predicted complexes of RPS2HD1 with ACP2-NOI and ACP3-NOI were superimposed based on the RPS2HD1 domain.
